# Stability of Sodium Dodecyl Sulfate Micelles in Reline/Water Mixtures: A Molecular Dynamics Study

**DOI:** 10.1021/acsomega.6c02912

**Published:** 2026-08-19

**Authors:** Rafaela Eliasquevici, Kalil Bernardino

**Affiliations:** Laboratório de Química Computacional, Departamento de Química, 67828 Universidade Federal de São Carlos, Rod. Washington Luiz S/n, 13565-905 São Carlos, Brazil

## Abstract

Deep eutectic solvents of type III, such as reline, a mixture of choline chloride and urea, are a relatively new class of green solvents that display physical properties intermediate between conventional salt solutions and ionic liquids. Well-established models used to explain colloidal stability and macromolecule conformation in usual solvents, including the hydrophobic effect and the formation of the electrical double layer, are not applicable to these solvents and their mixtures with water due to strong nonideal behavior and high ionic strength of those solvents. Hence, in this work, molecular dynamics simulations were performed to evaluate the thermodynamic stability of sodium dodecyl sulfate micelles in reline/water mixtures with different compositions by potential of mean force calculations and splitting the contributions from the surfactant ionic head and the hydrophobic tail. The presence of reline reduces the favorable contribution of the surfactant alkyl tail to aggregation, indicating a reduction of the hydrophobic effect, which is explained by a smaller number of water molecules in contact with the surfactant. However, the high adsorption of choline cations over the micelle surface also reduces the repulsion between the charged heads. The second effect dominates over the reduction of the hydrophobic effect, resulting in a more negative aggregation free energy and improved micelle stability in the presence of reline. The electrostatic potential from the anionic micelle was also computed and does not follow the usual electrical double layer models, presenting oscillations away from the micelle surface in mixtures with high reline concentration and with the choline cations almost completely replacing the sodium counterions from the micelle surface.

## Graphical abstract

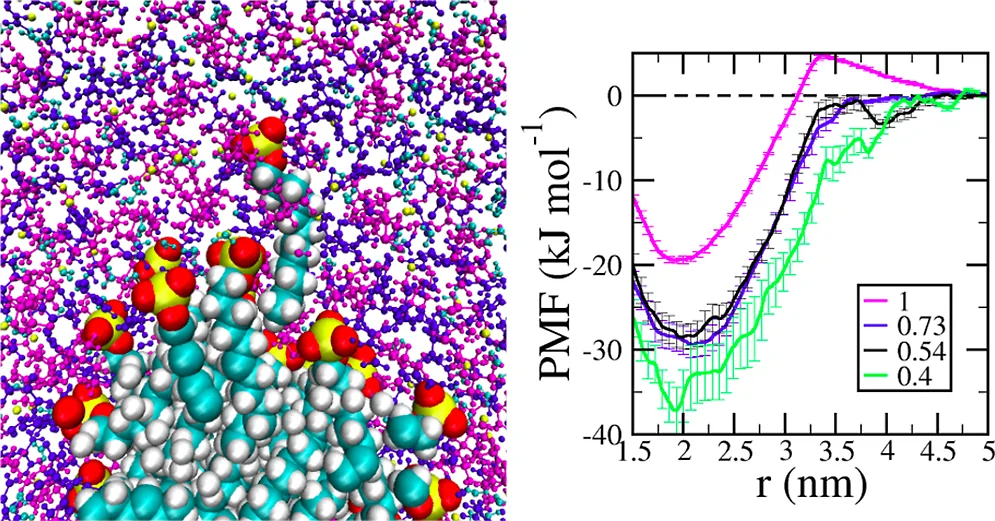

## 1 Introduction

Deep eutectic solvents (DESs) are a relatively new class of solvents, with initial studies dating from 2003,^1^ which consist of a mixture of a hydrogen bond donor and a hydrogen bond acceptor that results in a dramatic reduction of the melting point. The most studied DES, called type III DES, consists of mixtures of an ionic and a molecular compound, like the reline, which corresponds to the mixture of choline chloride and urea in the 1:2 molar proportion, and glyceline, which corresponds to the mixture of choline chloride and glycerol, also in the 1:2 proportion. Those solvents present physical properties intermediate between those of salt solutions and of ionic liquids since they present a very high ionic strength but are not composed exclusively of ions,^2^ presenting interesting physical properties of ionic liquids like negligible vapor pressure and high thermal stability, while also being considered nontoxic green solvents with lower production costs than ionic liquids.^3,4^

To efficiently explore the solvent capacities of DES for complex materials, including biomolecules^5,6^ and nanostructures,^7^ we need to better understand the molecular-level organization of DES around polar and apolar solutes and the thermodynamics involved in solvation. In water, hydrophobic forces and the formation of the electrical double layer are crucial for protein folding and stabilization of colloidal dispersions. Those forces are now well understood for aqueous solutions thanks to decades of experimental and computational studies. The hydrophobic forces are responsible for the segregation of apolar groups, such as alkyl groups, in order to reduce the number of water molecules in contact with the apolar group.^8^ Although the solvation of a small hydrophobic solute in water is exothermic, only some orientations of a water molecule in the first coordination shell of an apolar solute are possible in order to avoid the breaking of hydrogen bonds.^9^ Thus, the network of water molecules around an apolar solute or group is more organized than in the bulk, leading to an unfavorable entropy reduction. The segregation of those apolar groups due to either a phase separation, the formation of clusters like micelles, or the hindering of apolar amino acids inside the tertiary structure of a protein, releases the water molecules, which results in an entropy increase of the solvent, although the solute seems to evolve into a more organized structure. This effect was described first by Frank and Evans and was called the “iceberg model”.^10^ The increase of water organization and entropy reduction around hydrophobic solutes was confirmed by both computer simulations^11–13^ and scattering experiments.^14–16^

On the other hand, the formation of the electric double layer results in a long-range repulsion between charged particles and surfaces in aqueous solutions, which provides colloidal stability. In solutions with low ionic strength, well-known models are used to explain the formation of the electric double layer, like the Stern model, which considers that some counterions are bound strongly to the surface, subject to specific interactions and forming the Helmholtz plane,^17^ but the rest of the counterions are only weakly attracted, forming the diffuse layer, where the concentration of counterions decreases continually as moving further from the charged surface/particle and converges to the bulk concentration at large distances.^18,19^ When particles or surfaces with the same charge approach each other in an aqueous solution, a superposition takes place between the clouds of counterions in the respective diffuse layers, rendering a long-range repulsion.^17^

While those effects are now well understood for aqueous solutions, the same cannot be said about the DESs. The presence of larger molecules with groups not directly involved in hydrogen bonds may decrease the importance of the hydrophobic effect in those solvents, and the very high ion concentration breaks the hypothesis used to derive the electric double layer models in usual solvents. The aggregation of ionic surfactants into micelles is a good candidate to evaluate both the changes in the hydrophobic effect and ionic interactions due to the relatively fast relaxation and simple chemical structure when compared to a protein or a charged solid surface.

The stability of micelles in DES and in water/DES mixtures depends on the chemical composition of both the DES and the surfactant. One of the best indicators of micelle stability is the critical micellar concentration (CMC), the lowest concentration at which micelles are formed, since a smaller CMC corresponds to a more negative aggregation free energy. The CMC of dodecyl sulfate surfactant with several different counterions indicates that the stability of their micelles increases on moving from water to glyceline and to reline.^20^ The sodium dodecyl sulfate (SDS) CMC, for instance, is 8.1 mmol/L in water, 3.9 mmol/L in glyceline, and 2 mmol/L in reline at 25 °C.^20^ The micelles observed in DES are also much larger, as the SDS aggregation number increases from 50 ± 4 in aqueous solution at the CMC^21^ to 781 ± 70 in reline^22^ at a similar concentration. Also, the shape of the SDS micelles changes from spherical to elongated when moving from water to reline, an effect that is attributed to a reduction of the repulsion between surfactant headgroups, and the addition of water to reline decreases the SDS aggregation number.^22^ Considering reline/water mixtures, fluorescence probe measurements indicate that the CMC behavior is nonmonotonic, with the addition of 5% in weight of water to reline leading to a larger increase in the CMC, while the addition of 15% or 50% of water decreases the CMC again.^23,24^ However, the diameter of the aggregates systematically decreases with the addition of water, and both the specific conductivity and surface tension measurements of the same authors suggested much smaller sensitivity of the CMC with water addition to reline than the fluorescence probe determinations.^24^ Smaller CMC and large aggregation numbers were also reported for cationic surfactants in glyceline and ethaline than in water.^25,26^ Zwitterionic amphiphiles, however, display the opposite trend, with sulfobetaine and phosphocholine-based surfactants displaying CMC values ca. 8 times higher in glyceline than in water.^27,28^

Similarly to glyceline, ethaline, and reline, DESs formed by mixtures of lanthanide nitrates [M­(NO3)­3·6H2O], where M = La or Ce, with urea result in a decrease of CMC values for the ionic surfactants SDS, DTAC, DTAB, and C12TANO3, but in an increase of CMC values for nonionic surfactants C12EO6, C12EO8, and TX-100.^29,30^ Also, increasing the urea fraction in those DES leads to a significant CMC decrease for ionic surfactants but to a small increase for the neutral surfactant TX-100.^30^ Those results indicate that the screening of the ionic repulsion between charged heads in ionic surfactants due to the high ion concentration in the solvent is a key feature to increase the stability of ionic surfactant micelles, but the same effect is absent for neutral surfactants, for which the smaller solvophobic effect leads to a smaller micelle stability.

Although the cluster size and CMC data give useful insights into the effect of DES on intermolecular forces involved in surfactant self-assembling, they cannot probe the molecular-level solvent structure and quantify how the specific solvent–solute interactions modulate the self-assembling. To fill this gap, computer simulations can provide a detailed picture of the DES organization around self-assembled structures, providing molecular-level information necessary for the development of new models to explain the contributions of solvophobic effects and ionic interactions in DESs and DES/water mixtures. Despite several computer simulations having been performed in the last 20 years to study physical properties of DESs^31^ and their mixtures with water,^32,33^ there are still only a few simulation works focusing on solute self-assembly in DES. Some recent works include the study of ionic micelles in pure DES,^34^ the formation of small clusters of heteroaromatic drugs in reline,^35^ and the influence of reline on conformational changes of proteins^36,37^ and DNA quadruplex.^38^ Besides those, some works also focused on the individual effects of urea or choline chloride on the aggregation of amphiphiles or hydrophobic solutes in aqueous solutions. Molecular dynamics simulations of the spontaneous self-assembly of sodium octanoate reveal the formation of smaller micelles and a larger concentration of free surfactant monomers in water/urea mixtures than in pure water, displaying a reduction of the hydrophobic effect due to the preferential solvation of the hydrophobic tail by urea than water molecules, especially considering the solvation of CH_2_ groups farther from the polar head.^39^ On the other hand, the addition of choline chloride to water was found to increase the hydrophobic effect, resulting in a more negative association free energy between methane molecules in choline chloride-concentrated solutions than in pure water.^40^ Hence, urea and choline chloride seem to display opposite effects regarding hydrophobic aggregation when added separately to water, and further studies are necessary to understand their combined effects in reline/water mixtures. In this work, we will study the aggregation of the anionic surfactant SDS into micelles in pure water and in mixtures of water with the DES reline at different ratios between the two solvents in order to understand the effects of the DES over both ionic and hydrophobic interactions by means of performing both equilibrium simulations and potential of mean force (PMF) calculations.

## 2 Methods

### 2.1 Model System Preparation and PMF Calculation

In order to study the aggregation thermodynamics of SDS micelles in reline/water mixtures, both equilibrium simulations and potential of mean force (PMF) calculations were performed. For comparison and validation purposes, the SDS micelle was studied in both pure water and in reline/water mixtures with 3 different ratios. The initial structure for the micelle in pure water simulation was generated using the PACKMOL program,^41,42^ so the surfactant heads point outward from a sphere of radius 1.4 nm and the terminal methyl groups were placed inside a sphere of radius 0.85 nm. Subsequently, 42,000 solvent molecules and 60 counterions were added to fill a cubic box with an edge length of 16 nm, corresponding to a surfactant molar concentration of 24 mmol/L, above the experimental CMC in both water and reline. For the simulations in reline/water mixtures, the equilibrated structure of the SDS micelle obtained in pure water was extracted and introduced into a new cubic box filled with solvent molecules and ions using PACKMOL, with 6820 urea molecules and 3410 choline chloride ion pairs being added in each system. The different dilutions were made by adding different amounts of water molecules in the mixtures: 6820 (1 per urea molecule), 13,680 (2 for each urea), and 27,280 (4 for each urea). Those systems will be referred to based on the resulting water mole fractions in the solvent in this manuscript, which are 0.40, 0.54, and 0.73, respectively.

To evaluate the solvation of a simple hydrophobic solute, simulations of a single methane molecule in water and reline/water mixtures with the same molar fractions employed for the micelle were also performed. For pure water, a box with 1 methane and 2838 water molecules was produced using the gmx solvate tool of Gromacs, while for reline/water mixtures, the initial structures were generated using PACKMOL by adding 1 methane molecule, 200 choline chloride ion pairs, 400 urea molecules, and 400, 800, and 1600 water molecules to generate the water molar fractions in the solvent of 0.40, 0.54, and 0.73, respectively. 60 ns simulations were performed for each composition except for the system with the lowest water content, for which a 100 ns simulation was performed.

To compute the aggregation-free energy in each system, PMF calculations were performed using the umbrella sampling method. For this purpose, a dodecyl sulfate ion from the micelle (monomer) was dissociated along the reaction coordinate *r* between the micelle center of mass and the sulfur atom of the surfactant head. This method involves applying a harmonic potential (eq 1), known as the bias potential, in addition to the forces typically present in the system.

1 $$U_{bias}\left(r\right)=\frac{1}{2}k{\left(r-r_{\min}\right)}^{2}$$

Successive simulations were performed by varying the minimum position *r*_min_ of this potential every 0.1 nm, starting at 1.5 nm, slightly before the maximum of the radial distribution of the surfactant head relative to the micelle center of mass (Figure S1 in Supporting Information), up to 5.0 nm, thus forcing the monomer to span the entire reaction coordinate toward dissociation, as shown in Figure 1.

**Figure 1:**
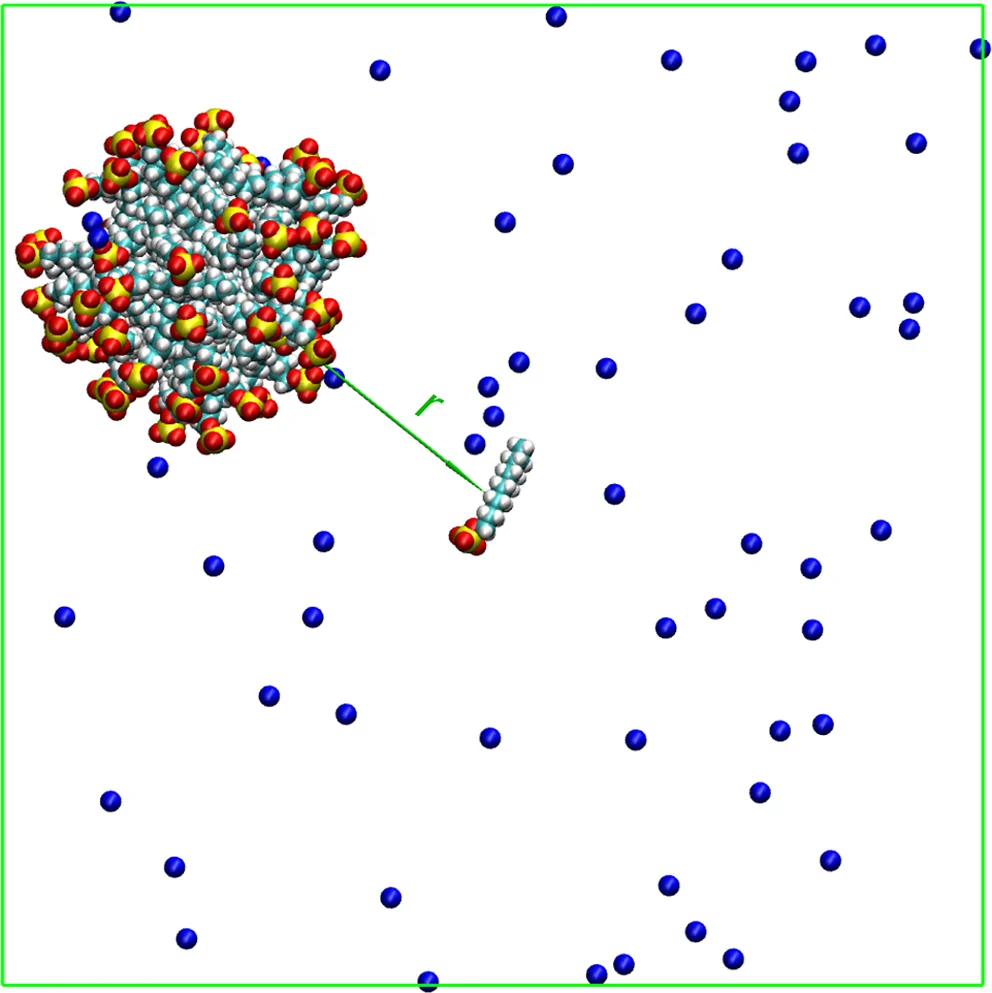
Representation of a monomer being pulled from the micelle in the simulation box with the following colors: hydrogen (gray), carbon (cyan), oxygen (red), sulfur (yellow), and sodium (blue). The solvent has been hidden for better visualization. The green vector represents the reaction coordinate, extending from the micelle center of mass toward the pulled monomer.

Further PMF calculations were carried out to remove the surfactant headgroup, with the aim of understanding the contributions of the head and tail in thermodynamics. The headgroup was modeled as a methyl sulfate ion, which was obtained by converting the alkyl chain of one of the dodecyl sulfate ions present in the micelle into a CH_3_ group. The choice of the methyl sulfate group over sulfate or hydrogen sulfate was more appropriate, as those alternatives would introduce hydrogen bonds or charges not originally present in SDS. A similar choice was used in a previous work to separate the head and tail contributions for sodium octanoate micelles, in which the polar headgroup was modulated as an acetate anion.^46^ Furthermore, in the force field employed, the partial charge of the carbon atom adjacent to the headgroup differs from that of the other tail carbons (Table S1). Consequently, this first group should be considered to be part of the surfactant headgroup rather than the tail. To this end, these PMF calculations were carried out for the aggregates in pure water and an intermediate reline/water mixture with a water mole fraction of 0.54.

To calculate the PMF, the probability distributions obtained in the various simulations were combined using Weighted Histogram Analysis Method (WHAM)^43^ implemented in the GROMACS software (version 2021.4),^44,45^ providing the free energy of aggregation and information on energy barriers for this process. To ensure the quality of the PMF, all distances along the reaction coordinate must be adequately sampled, showing good overlap between neighboring sampling histograms. The force constant value of 1000 kJ mol^–1^ nm^–2^, already used in previous works,^46^ proved suitable since the requirement of obtaining good overlap between histograms in the simulations was met, as can be observed in Figure S2 of Supporting Information.

### 2.2 Simulation Conditions and Parameter

The study was conducted through classical molecular dynamics simulations. All simulations were performed using the GROMACS suite (versions 2020.4 and 2021.4),^44,45^ with an integration time step of 0.8 fs. Before starting the molecular dynamics simulations, an energy minimization was performed using the steepest descent algorithm. The structural relaxation was determined by the convergence of the system’s density and counterion adsorption on the micelle surface. For the equilibrium systems with water molar fractions of 0.40, 0.54, 0.73, and 1, the total simulation times were 480, 384, 472, and 480 ns, respectively. For the PMF, the total time integration was 16 ns for the micelle in water and 12 ns for the other systems in each sampling window along the PMF calculation. In the simulation with the highest reline amount (water molar fraction of 0.4), PMF relaxation issues occurred due to its slow dynamics. Therefore, the calculation was extended by an additional 12 ns in each sampling window, totaling 24 ns per window. However, only those extended portions of the simulations were used.

All simulations were performed in the *NPT* ensemble, with temperature coupled to the V-rescale algorithm,^47^ using a bath temperature of 323.15 K and a coupling constant of 0.1 ps. The choice of a temperature above room temperature was made to ensure faster system relaxation, given the high viscosity of reline^48^ and the reline/water mixtures. The isotropic Berendsen pressure coupling^49^ was used with *P* = 1.0 bar and a coupling constant equal to 1.0 ps. Periodic boundary conditions were applied in all directions. The potential energy for dodecyl sulfate and choline ions was described using OPLS-AA parameters,^50^ with recalculated charges for dodecyl sulfate.^19^ The flexible SPC model^51^ was used for water molecules, and Åqvist parameters^52^ were applied for sodium cations. Nonbonded interactions were truncated at 1.2 nm, with Particle Mesh Ewald correction for long-range Coulomb interactions and a shifting potential starting at 1.0 nm to bring Lennard–Jones interactions smoothly to zero at the cutoff radius. No charge scaling was used in those simulations. The values for partial charges and Lennard–Jones parameters are given in Tables S1–S6 in the Supporting Information file. The visualization of trajectories and rendering of graphical representations of the systems were performed by the VMD program (version 1.9.4).^53^

## 3 Results and Discussion

### 3.1 Micelle Structure and Thermodynamics of Aggregation

No dodecyl sulfate dissociation was observed in the equilibrium simulations, with *N* = 60 micelles remaining stable for each solvent composition tested, although significant counterion exchange was noticed in the presence of reline, as will be discussed later. In order to evaluate possible changes in the micelle size and shape, solvent accessible surface area and the inertia moment components (*I*_*j*_) of the aggregates are computed according to eq 2, where *m*_*i*_ is the mass of each atom, and *r_ij_* is the distance between the atom and the reference inertia axis that passes through the structure center of mass. For a perfect sphere, all principal components of the moment of inertia should be identical, reflecting the uniform mass distribution in all directions. However, as shown in Table 1, the values of *I*_1_, *I*_2_, and *I*_3_ vary across the different composition systems, indicating that the micelles deviate from a perfect spherical shape. Consequently, we calculated the eccentricity (as defined in eq 3, where *I*_aver_ represents the average of the three moment of inertia components) for the aggregates, which provides a quantitative measure of how much the structure diverges from being spherical and approaches an ellipsoidal shape. Although not equal to zero, the eccentricity values are very small, indicating that the deviation from a spherical shape is minimal across all systems, which is consistent with the visual inspection of the structures presented in Figures 1 and 4.

2 $$I_{j}=\sum_{i}m_{i}r_{ij}^{2}$$

3 $$e=1-\frac{I_{1}}{I_{aver}}$$

**Table 1:** Structural and Thermodynamic Properties of the Micellar Systems as a Function of the Water Molar Fraction in the Solvent^a^

water molar fraction	dodecyl sulfate solvent-accessible surface area (nm^2^)	inertia moment (10^4^ au nm^2^)			eccentricity	Δ*G* aggregation (kJ mol^–1^)
		*I*_1_	*I*_2_	*I*_3_		
1	109.3 ±3.1	2.55 ± 0.12	2.96 ± 0.14	3.23 ± 0.15	0.13 ± 0.04	–19.5 ± 0.3
0.73	109.9 ± 2.8	2.55 ± 0.11	2.93 ± 0.13	3.21 ± 0.14	0.12 ± 0.04	–29.4 ± 1.6
0.54	108.4 ± 2.5	2.55 ± 0.11	2.90 ± 0.11	3.15 ± 0.12	0.11 ± 0.04	–29.6 ± 1.4
0.40	106.8 ± 2.5	2.51 ± 0.09	2.86 ± 0.11	3.10 ± 0.11	0.11 ± 0.04	–37.2 ± 2.6

a reported values include the solvent-accessible surface area, principal moments of inertia, eccentricity, and aggregation free energy. Values are given as mean ± standard deviation.

Additionally, comparison among systems shows no significant differences in aggregate shape across compositions. Both the moment of inertia components and eccentricity values are very similar, with a slight decrease observed as the reline concentration increases as well as the surface area. It is worth noting that, experimentally, micelles in reline tend to be larger, as reported by Arnold et al..^54^ However, since we are modeling only a single aggregate with a limited number of monomers, this effect is not fully captured in our simulations. Model systems with large amounts of dodecyl sulfate would be needed to observe the aggregate growth in the presence of reline.

We carried out PMF calculations to elucidate the thermodynamic and free energy profiles of dodecyl sulfate micelle aggregation in DESs (Figures 2 and 3). The PMF was conducted for the removal of a monomer of the aggregate in different reline/water compositions. The minimum corresponds to the surfactant head located at the aggregate surface, approximately *r* = 2 nm for all simulations. This is observed in the radial distribution function (RDF) *g* (*r*) of the sulfur atoms in the micelle headgroups relative to the micelle center of mass (Figure S1). Since the micelles exhibit approximately the same shape regardless of composition, this minimum appears at a similar radial position in all cases. The free energy difference between the PMF minimum and the dissociated state at *r* = 5.0 nm was taken as the aggregation free energy, Δ_ag_ *G* (Table 1).

**Figure 2:**
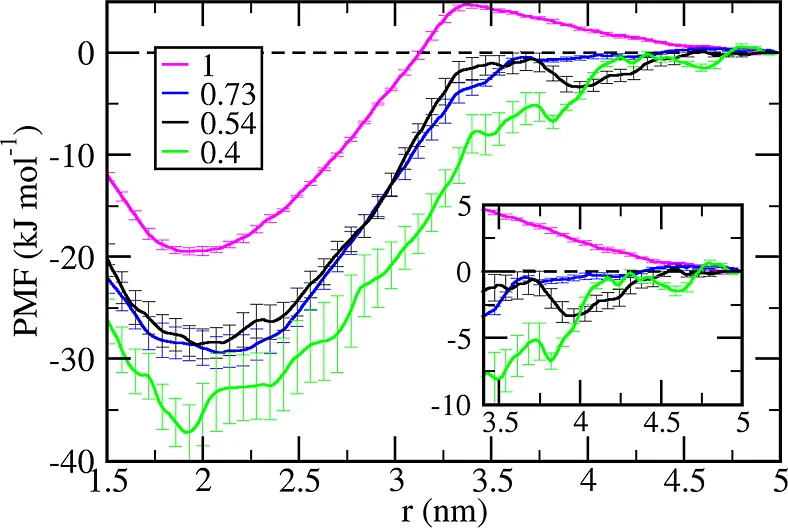
PMF for the removal of a dodecyl sulfate ion from the micelle. Each curve color corresponds to a different water molar fraction in the solvent. Inset show the behavior at long distances. Error bars were computed by the bootstrap method by dividing the data into 5 sets to compute the PMF and calculating the standard deviation among them (Figure S3 in the Supporting Information file).

**Figure 3:**
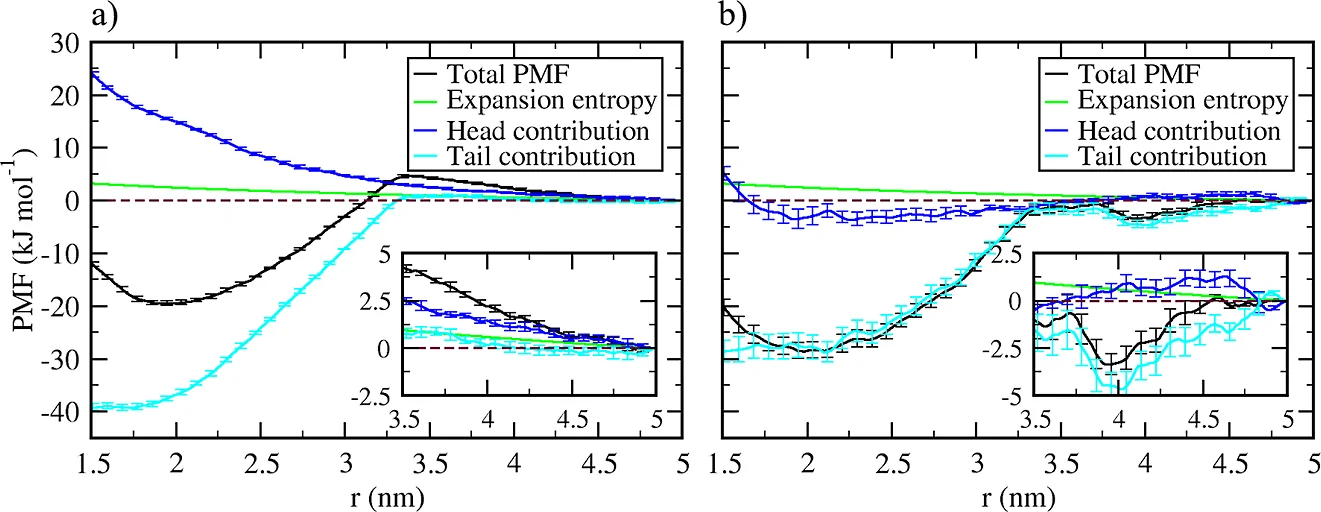
Estimation of surfactant head and tail contributions for the PMF computed by computing PMF profiles for the removal of a methyl-sulfate from the micelle for systems with water molar fractions of (a) 1 and (b) 0.54. Insets focus on the behavior at long distances. Error bars for total PMF and head contribution were computed by the bootstrap method by dividing the data into 5 sets to compute the PMF, while the error for the tail contribution was estimated using the error propagation formula (Δ*x*_1_^2^ + Δ*x*_2_^2^)^0.5^, where Δ*x*_1_ and Δ*x*_2_ are the errors for the total PMF and for the head contribution.

Moving to smaller distances *r* forces the surfactant headgroup to penetrate the micelle hydrophobic core, while moving toward larger *r* increases the hydrophobic tail exposition to water. Both situations lead to an increase in the Gibbs free energy. When the pulled dodecyl sulfate is completely removed from the micelle, which happens at nearly *r* = 3.3 nm for every solvent composition, the PMF reaches a maximum and, when moving to larger distances, only smaller changes are noticed in the PMF, which are due to the electrostatic potential generated by the micelle and entropic effects that will be discussed later. The difference between the free energy at the minimum and the largest distance sampled (*r* = 5 nm) will be taken as the aggregation free energy (Δ_ag_ *G*) (Table 1).

Notably, in reline/water systems, the Δ_ag_ *G* values are considerably more negative than in pure water. At intermediate reline concentrations (water molar fractions of 0.73 and 0.54), even when water is present in equal or greater amounts, the free energy decreases by approximately 10 kJ mol^–1^. In the system with the highest reline content, the Δ_ag_ *G* value decreases further, by about 18 kJ mol^–1^. These results indicate that SDS aggregates are significantly more stable in the presence of reline, which is consistent with experimental results that display both a CMC decrease and an aggregation number increase when increasing the reline molar fraction in SDS solutions. It is worth noting that these systems exhibit oscillations after the maximum at *r* = 3.3 nm, becoming more pronounced as the reline concentration increases. Those oscillations are related to electrostatic effects that will be discussed in Section 3.2.

To distinguish the tail and head contributions to micellar stability, we performed PMF calculations for surfactant head removal in pure water and at an intermediate reline concentration (water molar fraction of 0.54). As presented, the driving force for micelle aggregation arises from a balance between the hydrophobic effect, which tends to minimize solvent contact with the apolar tails, and the repulsive nature of the headgroups, which tends to increase their exposure to the solvent. Experimentally, it is well-known that these two contributions are nearly additive to the aggregation free energy at least in water.^17^ Thus, by subtracting the head PMF from the total PMF, we obtained the estimated tail PMF. Furthermore, the expansion entropy term (eq 4), intrinsic to the system, was removed from both head and tail contributions. This contribution is independent of the intermolecular interactions in the system (hence, independent of the solvent composition) and is a consequence of describing the PMF as a function of a radial coordinate, as moving from a spherical shell of radius *r*_*1*_ to a larger spherical shell of radius *r*_*2*_ leads to an increase of the accessible volume of 4π­(*r*_2_^2^ – *r*_*1*_^2^)*dr*, where *dr* is the thickness of the spherical shells. This contribution favors the dissociation and ultimately leads to micelles becoming unstable at very dilute systems (surfactant concentration smaller than the CMC).^55^

4 $$\Delta G=-2RT\cdot \ln\left(\frac{r}{r_{0}}\right)$$

For the micelle in pure water, the headgroup contribution to the overall stability is positive (blue curve), indicating an unfavorable effect on aggregation, as expected for an anionic surfactant. In contrast, in the reline/water mixture, the headgroups no longer contribute unfavorably. Instead, they exhibit a small negative free energy close to the micelle surface, thereby favoring micellar aggregation. Moreover, the tail contribution (cyan curve) in the reline/water system is less negative, indicating a reduction in the hydrophobic effect. Still, the stabilizing effect of the headgroups is substantially greater than the reduction of the hydrophobic effect such that the presence of reline ultimately leads to more stable anionic micellar aggregates.

### 3.2 Micelle and Dissociated Monomer Solvation

The first solvation shell around the SDS micelle at each solvent composition was analyzed based on a distance criteria, where we consider a molecule or ion as being in the first coordination shell of either the micelle or a dodecyl sulfate anion if any atom of the molecule or ion is located at 0.4 nm or less from any dodecyl sulfate atom. Figure 4 bottom displays the molecules and ions in the first coordination shell around the micelle at the final structures for the reline/water mixtures, while Table 2 contains the average number of each species in the first coordination shell around the micelles after relaxation. Radial distribution functions between the center of mass of the micelle and oxygen and nitrogen atoms in urea and choline as well as the oxygen atom in water, were also calculated to evaluate the component distribution in different simulation systems (Figure 5). It was observed that urea and choline penetrate the micelle core more deeply than water in the reline systems. It is also noted that nitrogen atoms penetrate slightly more than oxygen atoms for all compositions with reline. However, the oxygen atoms from urea show a higher peak for all reline/water compositions, despite the shift relative to urea, which is consistent with a greater number of urea molecules than choline cations around the aggregate. Notice also that atoms of urea and choline present a density increase near the aggregate surface, as can be inferred by the *g*(*r*) curves reaching maximum values above 1, while the water oxygen curve arrives at a plateau at *g*(*r*) = 1, indicating preferential interaction with the DES species in comparison with water. The cumulative number (CN) of water molecules as a function of the distance of the micelle center of mass and the density of solvent molecules and ions, without the normalization of the RDF, can be observed in Figures S8 and S9 in the Supporting Information. It is noticeable that the penetration of water and urea molecules at distances smaller than 1.5 nm; however, the penetration at 1.0 nm becomes an extremely rare event; hence, both water, urea, and choline cannot penetrate easily the hydrophobic core of the micelle.

**Figure 4:**
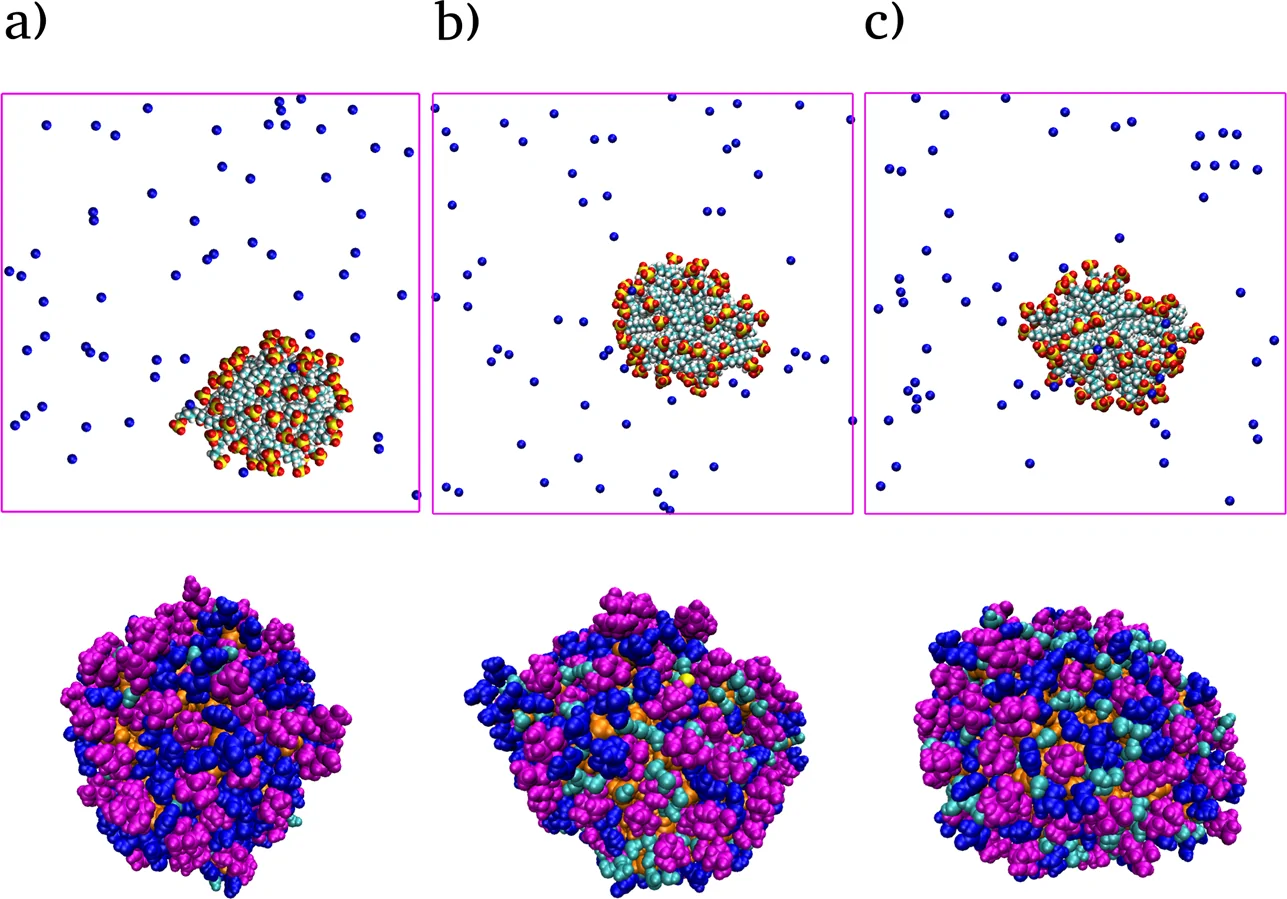
Graphical representations of the final structures of systems with water mole fractions of (a) 0.40, (b) 0.54, and (c) 0.73. Top: simulation boxes showing only the surfactant and the counterions with the following colors: hydrogen (gray), carbon (cyan), oxygen (red), sulfur (yellow), and sodium (blue). Bottom: first layer of molecules surrounding the micelle with the following colors: choline (magenta), urea (blue), water (cyan), chloride (yellow), and dodecyl sulfate (orange).

**Table 2:** Water Molar Fractions in the Systems along with Time-Averaged Corresponding Water Molar Fraction at the Micelle Surface and Number of Molecules of Each Component in the First Solvation Shell of the Aggregate^a^

water molar fraction in the system	water molar fraction in the first solvation shell of the aggregate	number of molecules in the first solvation shell of the aggregate				
		sodium	water	urea	choline	chloride
1	1	14.4 ± 3.0	894.8 ± 50.0	-	-	-
0.73	0.52	0.1 ± 0.3	285.3 ± 20.3	164.4 ± 11.2	92.4 ± 4.1	1.1 ± 1.0
0.54	0.37	0.1 ± 0.2	171.3 ± 15.5	179.7 ± 10.1	108.5 ± 4.5	2.0 ± 1.4
0.40	0.24	0.0 ± 0.0	94.8 ± 13.1	186.6 ± 9.1	117.9 ± 4.3	2.8 ± 1.7

a Values are given as mean ± standard deviation.

**Figure 5:**
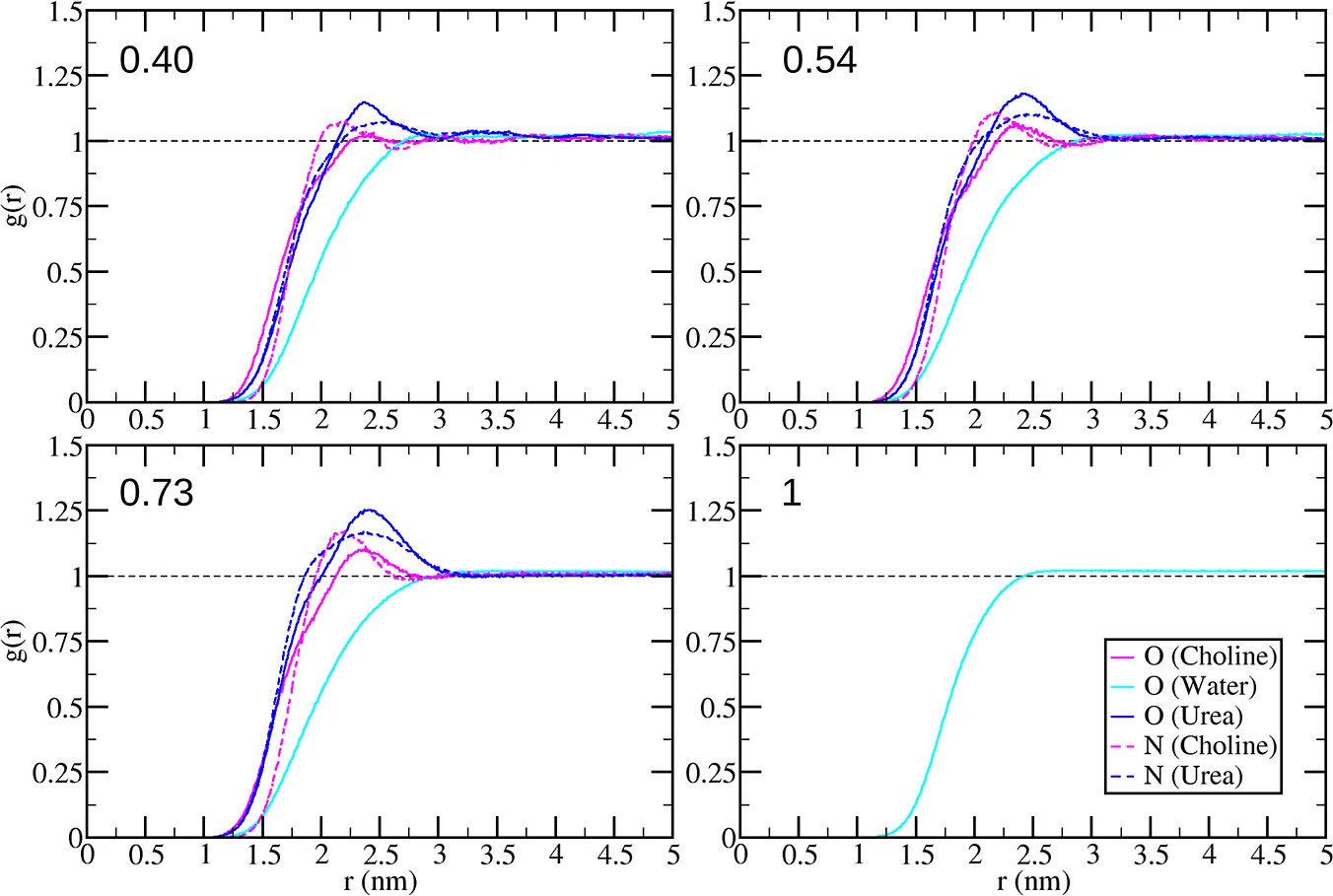
RDF between the micelle center of mass and the oxygen (solid lines) and nitrogen (dashed lines) atoms belonging to the groups of the systems.

As can be seen, the sodium ion, the natural counterion of dodecyl sulfate, is absent from the micelle’s first solvation shell in the final structure of the reline/water mixtures, being instead dispersed in the bulk solution, as noticed in Figure 4 (top). In contrast, a large amount of urea and choline interacts with the aggregate. For the systems with water molar fractions of 0.73 and 0.54, the average number of sodium ions in the aggregate solvation shell was 0.1. In the system with a water molar fraction of 0.40, this value decreases to essentially zero (computed value smaller than 0.02). Analysis of the trajectories showed that sodium only sporadically approached the micelle surface, remaining mostly in the bulk phase. In contrast, in the pure water system, approximately 14 sodium ions were located at the micelle surface. Although this number is relatively small compared to the 60 counterions present, the near absence of sodium in the reline-containing systems is significant. High amounts of urea and choline were found associated with the aggregate, and their populations increase with increasing reline concentration. Even though only a very small number of chloride ions interact with the micelle, an increase in their population is evident with increasing reline concentration, rising from approximately 1 to 3 chloride ions at the micelle surface. Previous work from our group has shown that the choline cation has a greater tendency to adsorb at the micelle surface than sodium, as evidenced by quantitative analysis based on counterion association numbers in SDS and choline dodecyl sulfate (ChDS) micelles in aqueous solution.^56^ In this work, we observed that choline interacts preferentially with the micellar aggregate not only when it acts as the counterion but also when it is present in the solvent and competes with sodium. In both situations, choline shows a stronger affinity for the aggregate than the sodium cation.

The almost complete absence of sodium at the micelle surface also noticed by the RDF, *g*(*r*) of Na^+^ around micelle center of mass (Figure S1 in Supporting Information file) and around the oxygen atoms of the surfactant headgroup (Figure 6), which displays not only a much smaller correlation between the surfactant headgroup and Na^+^ in the first coordination shell in the mixtures with reline (notice the pure water curve was multiplied by 0.1 for better visualization), but also a tendency of the Na^+^ to stay away from the micelle, with the *g*(*r*) increasing as moving away from the surfactant headgroup and converging to the bulk density only a few nanometers away—as can be seen in *g*(*r*) curves between the sodium and the center of mass of the aggregate (Figure S1 of Supporting Information). In pure water, the opposite trend is noticed with a very strong correlation close to the surfactant head and the sodium, followed by a decrease as moving away from the anionic group, as expected by usual models in conventional solvents. This result enforces that classic models, such as the electrical double layer, are inefficient at predicting ion distributions around aggregates in DESs, due to their complexity and high ionic strength. The same trend was observed in the *g*(*r*) between the sodium and the sulfur of the sulfate head (Figure S7 in the Supporting Information), so that, for reline/water mixtures, Na^+^ presents low organization around the aggregate, and the RDF increases toward the bulk. In the recent work of Voroshylova et al.,^34^ molecular dynamics simulations were performed for ionic micelles in pure DES, including reline, ethaline, and glyceline, and a different result was noticed, with a significant amount of sodium at the surface of SDS micelle in pure reline. However, they started with the Na^+^ ions close at the micelle surface and, due to the slow dynamics in the absence of water and at a lower temperature, it is likely that those simulations were not long enough to observe the counterion exchange. We performed a simulation of the SDS micelle in pure reline, but the results were not included in this manuscript since, even at 323.15 K, the dynamics of the surfactant and of the counterions are too slow, and they appeared frozen after a fast initial relaxation. Additional comments regarding the diffusion at different water molar fractions will be presented by the end of this section. Longer simulations and, most likely, with polarizable force fields, will be needed to properly describe the counterions dynamics around micelles in pure DES, but the extrapolation of our results suggests that at least simple monovalent cations like Na^+^ are almost completely replaced by the choline cation at the micelle surface.

**Figure 6:**
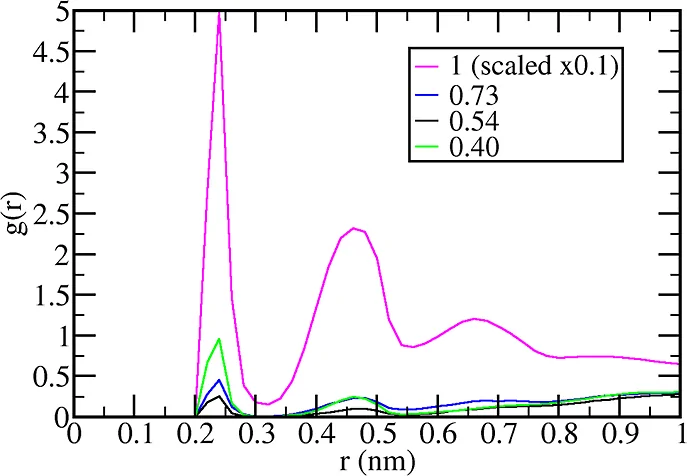
RDF of the sodium cation around the sulfate oxygen atoms of the surfactant headgroup for the equilibrium simulations of the micelles in water and reline/water mixture. For better visualization of the curves in the reline/water mixtures, the pure water curve was scaled by *a* factor of 0.1.

Water is also a key component of the analysis. Even at higher water molar fractions, where water is more abundant than reline, interactions of the surfactant with urea and choline appear to be dominant, indicating a preference for solvation by the reline components in comparison to that of water in the mixed solvent system. Although a significant reduction in the number of water molecules at the micelle surface was observed, the overall water content in the system decreased. Therefore, for comparison, the water molar fraction at the aggregate surface (Table 2) was calculated as the ratio between the number of water molecules at the micelle surface and the total number of molecules or solvent ions in the surface layer. In the mixture with a higher water concentration (0.73), the water molar fraction around the aggregate is approximately 29% lower than that in the bulk. This trend persists for the other compositions: the decrease is approximately 31% for the 0.54 system, and this value increases to about 40% for the 0.40 system, showing that the behavior of the solvent in these systems is far from ideal and indicating a preference for the reline instead of water close to the micelle surface.

We also calculated the number of species in the first layer of the dissociated monomer at different distances along the PMF calculation (Figure 7), which agrees with the presented conclusions. For this purpose, we chose relevant points along the reaction coordinate from the corresponding PMF profiles and calculated the average number of species present within a distance of 0.4 nm from any atom of the pulled dodecyl sulfate. As discussed before for the micelle surface, no appreciable sodium interaction was found with the monomer. Some amount of chloride ions appears in certain regions, particularly in systems with higher reline concentrations and away from the micelle, indicating that it can be present in the first coordination shell of the dissociated monomer, as also noticed by the structures in Figure 4. However, the large error bars demonstrate that the fluctuation of the Cl^–^ amount in the first coordination shell of the monomer is huge and the presence of Cl^–^ should be regarded as transient instead of resulting from a particular interaction with the monomer.

**Figure 7:**
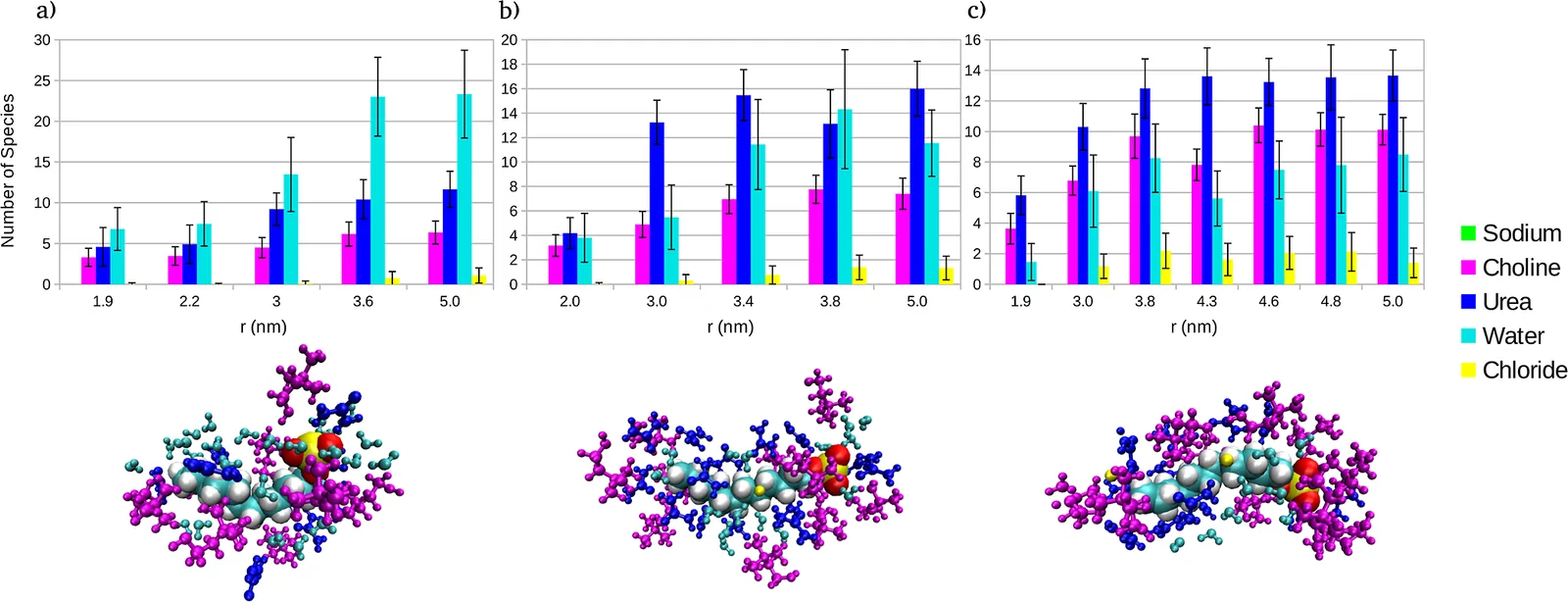
Top: time-averaged number of species in the first solvation shell of the monomer being pulled in different regions for simulations with water mole fractions of (a) 0.73, (b) 0.54, and (c) 0.40. Bottom: structures showing the first solvation shell of the monomer generated at *r* = 5 nm. Error bars correspond to the standard deviation of the number of molecules at different simulation frames.

In the system with a water molar fraction of 0.73 (Figure 7a), water is present in greater amounts than urea and choline around the monomer at all distances from the micelle center, with its quantity increasing as the dodecyl sulfate moves away from the micelle as its exposed area to the solvent also increases. In the system with a water molar fraction of 0.54 (Figure 7b), where reline and water are present in approximately equal amounts, the region near the micelle surface (*r* = 2.0 nm) exhibits a slightly higher amount of urea than water, along with a significant presence of choline. In the bulk region of this system, oscillations between water and urea quantities are observed, consistent with oscillations seen in the PMF curve. In the system with a water molar fraction of 0.40 (Figure 7c), the amount of water decreases further, while urea and choline remain prominently associated with the monomer. In this system, domain alternation after surfactant dissociation becomes more visible.

This result helps explain the decline in the tail contribution to aggregate stability, indicating that the hydrophobic effect is weakened in reline/water compositions compared to pure water. Otherwise, the large number of cations surrounding the micelle and the dissociated monomer, compared to sodium in pure water, dramatically changes the contribution of the anionic head to Δ_ag_ *G*, going from positive in pure water to slightly negative in systems with reline. This phenomenon is a consequence of the stabilization of the charged sulfate groups due to the high cation concentration at the micelle surface, favoring aggregation.

In order to understand how the mixture with reline affects the hydrophobic effect, the solvation of a single methane molecule was analyzed in systems with the same solvent compositions used for the micelles (Figure 8). When reline is introduced, part of the water molecules in the first solvation shell of methane are replaced by urea molecules and choline cations, and, since those molecules are bulkier than water molecules, the overall number of species around the methane molecule diminishes (Figure 8a). The average number of water molecules in the first coordination shell changes from 22.2 in pure water to 9.3, 6.1, and 3.5 for the mixtures with water molar fractions 0.73, 0.54, and 0.40, respectively (RDF of water oxygen around methane given in Figure S10 in the Supporting Information file). As a consequence, the number of water–water hydrogen bonds involving at least one molecule in the methane first solvation shell gets progressively smaller (Figure 8b and Table S10) and, even with the introduction of hydrogen bonds involving choline and urea, the overall number of hydrogen bonds close to the methane molecule decreases significantly in the mixtures with reline. The hydrogen bonds were analyzed using the Gromacs tool *gmx hbond*, which defines a hydrogen bond by geometric criteria, where the donor–acceptor distance must be smaller than 0.35 nm, and the hydrogen–donor–acceptor angle must be smaller than 30°.

**Figure 8:**
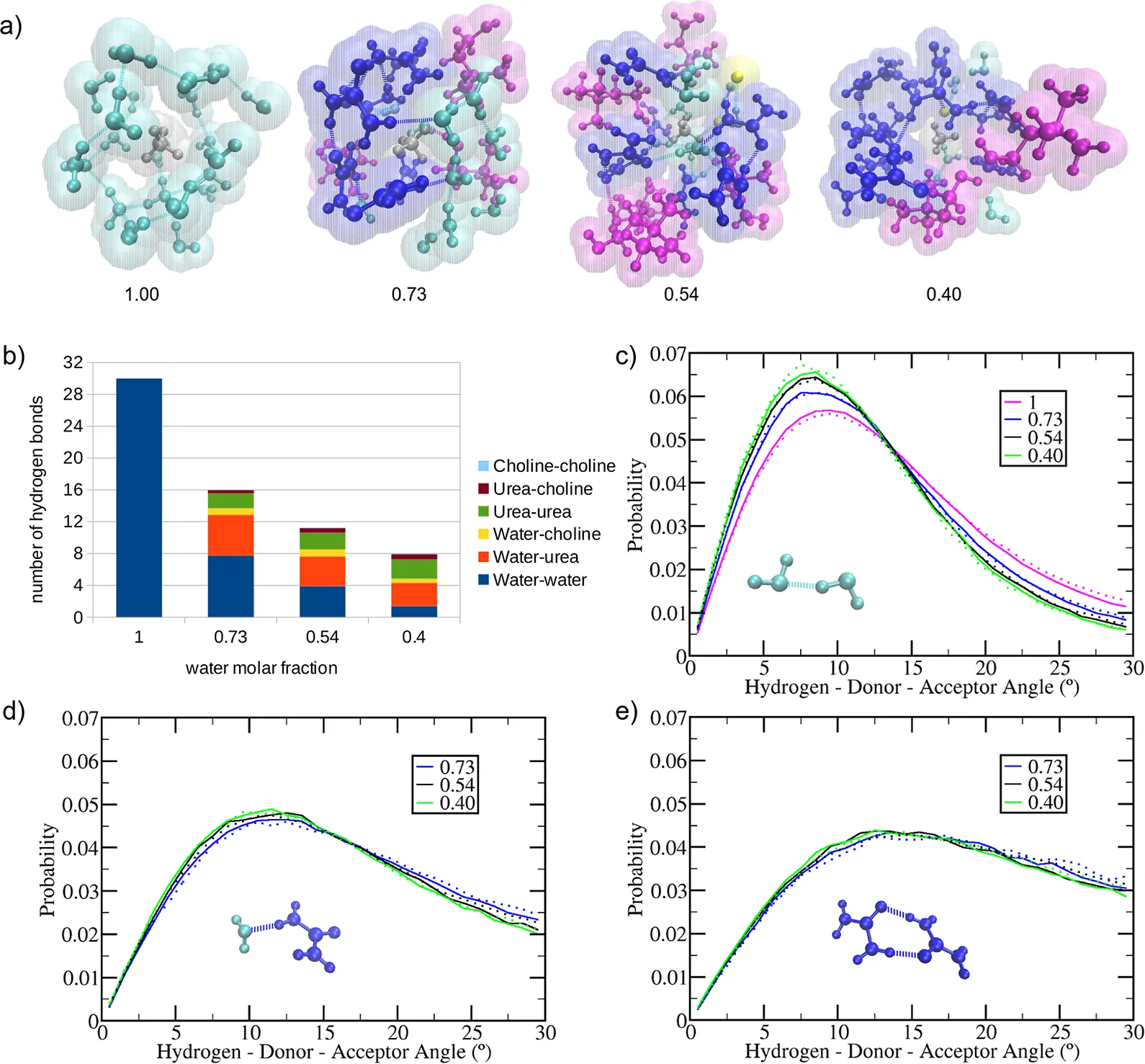
Solvation of methane in water and reline/water mixtures. (a) Solvent molecules in the first coordination shell of methane with the hydrogen bonds between them represented by dashed lines. Colors: choline (magenta), urea (blue), water (cyan), chloride (yellow), and methane (silver). (b) Average number of hydrogen bonds involving solvent molecules and ions in the first coordination shell of methane molecules. (c-e) Distribution of hydrogen–donor–acceptor angle for hydrogen bonds involving molecules of the methane first coordination shell (solid lines) and in pure solvents (dotted lines) for water–water (c), water-urea (d), and urea–urea (e) hydrogen bonds. Different colors stand for different water molar fractions given in the legends.

This effect alone would be enough to expect a reduction in the traditional hydrophobic effect due to the entropy reduction of the hydrogen bond network close to a hydrophobic solute, as noticed by the analyses of the hydrogen–donor–acceptor angle in pure water (pink curves in Figure 8c), which displays a slightly more concentrated distribution around the methane molecule (solid pink curve) than in pure water (dotted pink curve). In the presence of reline, on the other hand, the distributions around methane and in bulk solvents (compare solid with dotted curves for black, blue, and green curves) are more similar to each other than in pure water, indicating that the perturbation due to a small hydrophobic solute over water–water hydrogen bonds gets smaller. In fact, the reline itself induces larger changes in water–water hydrogen bond angles than the presence of the solute. In mixtures with reline, there are also a significant number of water-urea and urea–urea hydrogen bonds around methane, hence, the angle distribution for those hydrogen bonds was also analyzed (Figure 8d–e), but the angle distribution is broader than for water–water hydrogen bonds, and the urea–urea and water-urea angle distributions are not perturbed by the hydrophobic solute. In summary, the smaller hydrophobic effect in reline/water mixtures is due to three effects: an overall reduction in the number of hydrogen bonds close to the solute due to the introduction of bulkier solvent species, a reduction of the organization induced by the hydrophobic solute over the remaining water–water hydrogen bonds and to the fact that the hydrogen bonds involving urea are less organized and are not significantly affected by the solute, at least regarding the hydrogen bond angle distribution.

### 3.3 Electrostatic Potential and Solvent Orientation and Dynamics

We also calculated the total electrostatic potential (Figure 9) of the system as a function of the distance r from the micelle’s center of mass. Once partial charges had been assigned to all atom types in the simulation by the force field (Table S1-S6 of Supporting Information), we used the RDF of each atom type relative to the aggregate center of mass to compute the average charge density within spherical shells of radius *r*. Therefore, we computed the accumulated charge *Q*(*r*) from the integral of the charge density ρ­(*r*) (eq 5). We then grouped atom types by species and summed their contributions to obtain the total system accumulated charge (Figures S4 and S5 of Supporting Information). The electrostatic potential profiles were determined from the accumulated charge curves using Gauss’s law under the assumption of spherical symmetry (eq 6, in which Q­(r) is the accumulated charge inside a sphere of radius *r* centered on the micelle center of mass and ε_0_ is the vacuum permittivity). As discussed in Section 3.1, the aggregates exhibit an approximately spherical geometry. Additionally, the micelle rotates freely about all axes, resulting in a time-averaged shape that is even closer to spherical symmetry, ensuring the validity of the analysis. Our group has previously presented a similar analysis for related systems.^56^

5 $$Q\left(r\right)=2\pi \int_{0}^{r}\rho \left(r\right)r^{2}dr$$

6 $$\psi =\frac{1}{{4\pi \epsilon }_{0}}\frac{Q\left(r\right)}{r^{2}}$$

**Figure 9:**
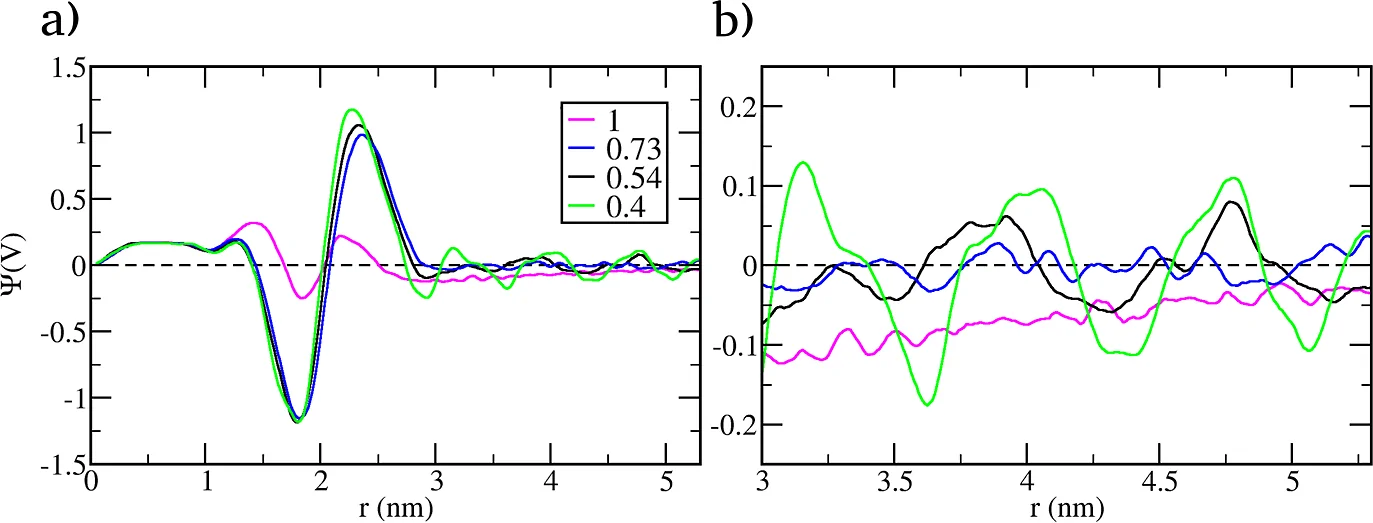
(a) Time-averaged total electrostatic potential as a function of the radial distance (*r*) from the micelle center of mass. (b) Zoom at long distances.

Within the aggregate, up to approximately *r* = 1.2 nm, the total electrostatic potential is essentially the same for all systems, since there is no solvent penetration inside the hydrophobic core of the micelle and also no significant structural change was induced in this region. In the very center of the micelle, the potential tends to zero because the accumulated charge also approaches zero as *r* → 0 (eq 5). Even so, the potential exhibits small positive values inside the hydrophobic core due to the contribution of positively charged aliphatic hydrogen atoms, which present a higher probability to be close to the micelle center than the aliphatic carbon atoms, which present negative partial charges, as noticed in previous works.^19,56^ Closer to the micelle surface, but still inside the aggregate, differences between the water and reline/water systems become apparent. Both systems shift toward positive values due to an increase in the positive contribution from water, as shown by the water electrostatic potential (Figure 10). However, because the pure water system contains a larger amount of water near the aggregate than the reline/water system, the rise of the total potential into the positive region is more pronounced, leading to a more intense peak.

**Figure 10:**
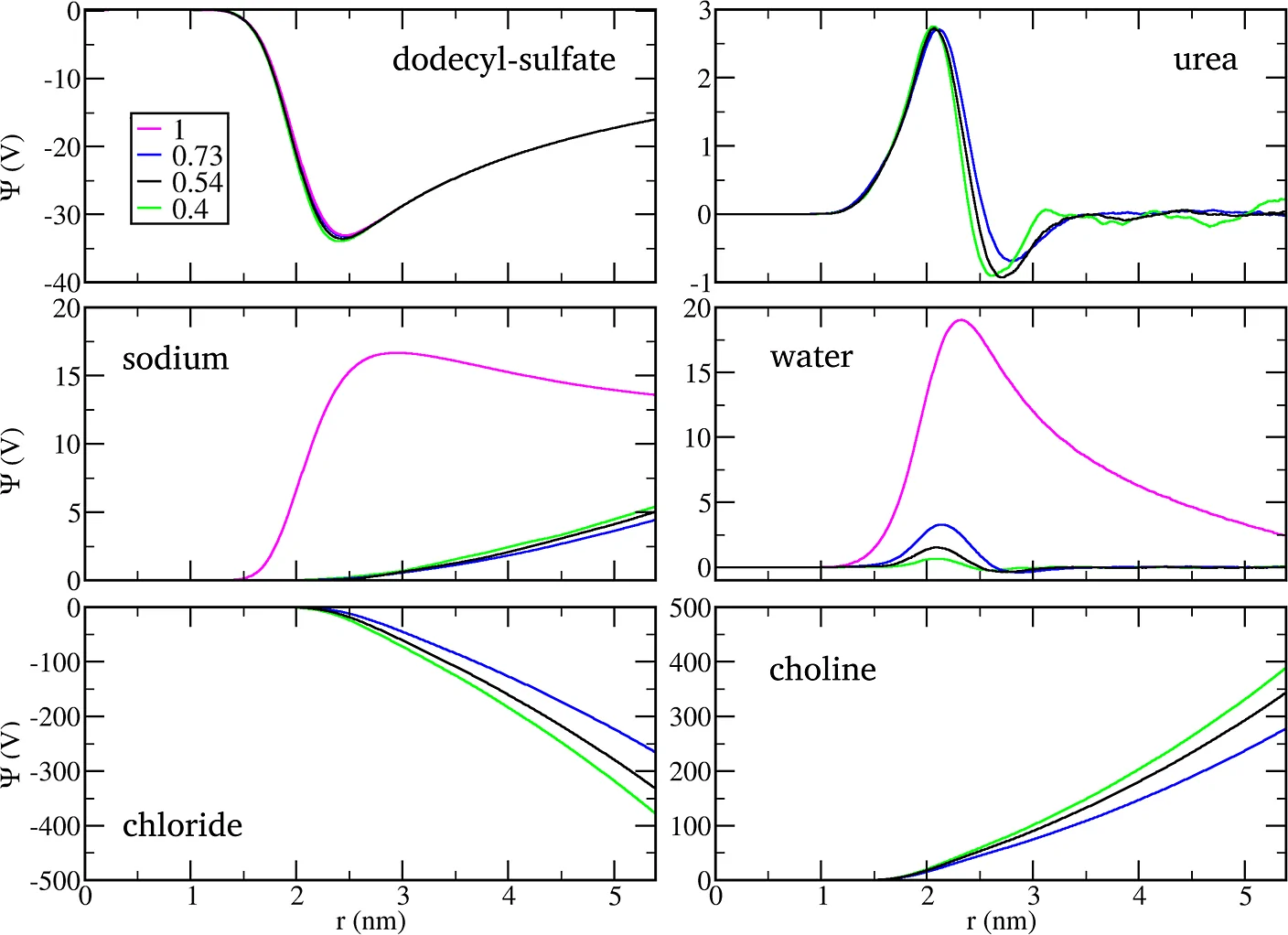
Contribution of each chemical species for the time-averaged electrostatic potential as a function of the radial distance (*r*) from the micelle center of mass for all system components. The corresponding accumulated charge profiles used to compute the electrostatic potential are given in Figure S5 in the Supporting Information file.

Even closer to the micelle surface, around *r* = 1.5 nm, the potential decreases significantly in all systems, as expected due to negatively charged headgroups (RDF in Figure S1). Nonetheless, the systems with reline present a peak at substantially lower values, which is a consequence of two effects: the lack of orientation (Figure 11) and less water near the aggregate surface (Table 2 and Figure 4). The water electrostatic potential profiles (Figure 10) show that, in systems containing reline, the curves are shifted toward larger distances and display lower intensity relative to the aggregate in pure water. The choline cations show a stronger association with the micelle headgroups than sodium ions, as observed in those systems by the average number (Figure 7) and by the oxygen–sodium RDF (Figure 6) and previously reported by our group by the comparison of ChDS and SDS micelles in aqueous solution.^56^ As a result, water penetration into the polar region of the micelle decreases. Additionally, the higher concentration of cations at the aggregate surface partially neutralizes the negative potential of the headgroups, thereby reducing the electrostatic driving force responsible for orienting water hydrogen atoms toward the micelle.

**Figure 11:**
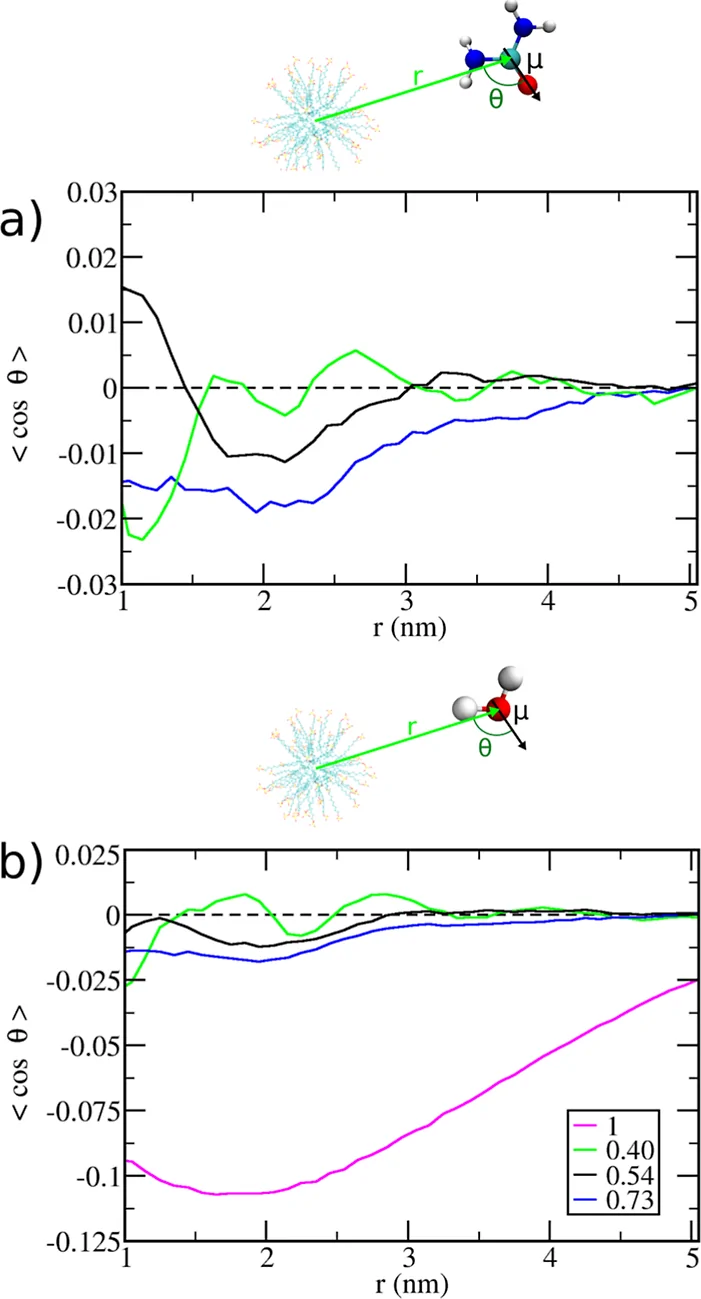
Time-averaged orientation of (a) urea and (b) water molecules at different distances from the micelle center of mass.

To compute this effect, we calculated the average orientation of water and urea relative to the aggregate center of mass. Specifically, cos­(θ) was defined as the cosine of the angle between the molecular dipole moment vector μ (which has the same orientation for both water and urea, as they belong to the same symmetry point group C_2_v) and the vector connecting the micelle center of mass to the oxygen atom of each molecule, as shown in Figure 11. By this approach, if the oxygen atom points toward the micelle, θ < 90°, resulting in positive cos­(θ) values. In contrast, when the hydrogen atoms of water molecules or the amino groups of urea molecules are directed toward the micelle, θ > 90°, leading to negative cos­(θ) values. An average value close to zero indicates no preferential orientation, as the number of molecules pointing toward and away from the micelle becomes nearly the same.

For the aggregate in pure water, the average cos­(θ) for water orientation is consistently negative (Figure 11b), indicating that the hydrogen atoms are preferentially oriented toward the micelle. The highest absolute values happen near the negative micelle surface, as expected. As the distance from the surface increases, the curve gradually approaches zero, indicating a progressive loss of preferential orientation of the water molecules. In reline systems, we observe that neither water nor urea exhibits a significant preferential orientation, as all curves, regardless of reline concentration, present average values of cos­(θ) closer to zero than in pure water. For the urea and water curves in the system with a 0.40 water molar fraction, the cos­(θ) values oscillate around zero, indicating that the high concentration of choline cations effectively neutralizes the negative micelle potential, such that even bulk molecules barely experience its electrostatic influence. For the other compositions, the curve profiles are predominantly negative, but, as mentioned, with minimum values of cos­(θ), which suggests that the hydrogen atoms and amino groups exhibit only a very weak preferential orientation. In our earlier work, we reported this behavior for water when comparing the electrostatic potentials and water orientation of SDS and ChDS aggregates, showing that the increase in the cation coordination at the micelle surface leads to a decrease in the water molecules’ orientation and in its contribution to the electrostatic potential.^56^ However, the higher choline concentration in the present systems leads to a more pronounced effect than noticed for ChDS in aqueous solution.

Notice, also, that water and urea present orientation within the distance range below 1.5 nm, which indicates that these species penetrate the micelle; however the hydrophobic core at distances smaller than 1.0 nm is essentially dry as discussed before (Figures S8 and S9 in Supporting Information file). This can be observed in Figure 5, which shows that urea and water *g*(*r*) curves start before the aggregate surface. Nevertheless, this was also quantified from CN curves (Figure S8 of the Supporting Information) and values at *r* = 1.5 (Table S8 of the Supporting Information), which confirms the presence of a significant amount of water and urea at distances smaller than 1.5 nm, although no significant penetration is noticed in below 1.0 nm. Table S9 reports the distances at which water and urea penetration become significant, using two criteria: CN and RDF values reaching 0.5. This first threshold corresponds to a 50% probability of finding a molecule within a given region and the second to the distance at which the water density reaches half of its bulk value at the given solvent composition. It should be noted that RDF values are normalized by the bulk density of each species, providing a more meaningful comparison between water and urea since they have different concentrations among the compositions. It is observed that, as we increase the reline concentration, both urea and water start to penetrate at longer distances, with urea penetrating more than water. For the CN criterion, the values are between 1.35 and 1.46 nm for both species in reline/water mixtures, but for the RDF criterion, it is observed that urea presents a deeper penetration (1.61–1.72 nm) than water (ca. 1.95 nm). This observation, together with the average numbers of these species at the aggregate surface—which shows that the number of water molecules decays significantly with the addition of reline, from almost 12 in the pure water system, to 3 in the mixture with the highest water molar fraction among the mixtures—aligns with the weakness of the hydrophobic effect in the presence of DES.

Returning to the total electrostatic potential, at *r* values outside the aggregate, the systems show the appearance of another peak, this time in the positive region. In reline/water systems, the maximum values are significantly higher due to an increase in cation concentration around the micelle. However, since the cations primarily accumulate on the surface of the aggregates, the electrostatic potential curves in the bulk region, in all systems, tend to approach zero. When zooming over the electrostatic potential curves at long distances (Figure 7b), a significant difference between the water and reline/water systems becomes evident. The total electrostatic potential in pure water always has negative values at long distances. Conversely, with the presence of reline, the total electrostatic potential fluctuates around zero in the same region, with its fluctuations intensifying as the reline concentration increases. As previously discussed, the increased cation concentration surrounding the micelle neutralizes the aggregate’s negative potential, thereby preventing its electrostatic influence from extending into the bulk region. This behavior explains why anionic micelles are known to be larger in the presence of reline compared to when they are in pure water. In the presence of reline, the micelles experience less repulsion due to a reduced negative potential in the surrounding medium. As a result, they tend to come closer together and merge, leading to an increase in their size and enabling the formation of elongated micelles.

When we analyze the other components of the electrostatic potential, we see an interesting behavior for sodium. In the pure water system, the curve begins to increase at approximately 1.5 nm, within the micelle, and reaches a maximum near the aggregate surface. The potential then decreases smoothly into the bulk of the simulation, which is expected for this system, following the electrical double layer model. When we analyze the sodium electrostatic potential curves in reline/water systems, we notice that the peak near the micelle surface disappears. As a result, the curves show a slightly linear increase toward the bulk of the simulation box. This pattern highlights the lack of classic electrical double-layer behavior in anionic micelles in DESs. In the context of the other ions present in the reline systems, choline and chloride show nearly the opposite trends in their electrostatic potential. Both species demonstrate similar growth in modulus with the slope of the curve increasing as the reline concentration rises. As for the potential of the dodecyl sulfate ion, it remains relatively constant across different compositions, starting near zero and achieving a negative peak near the micelle surface (at ca. *r* = 2.4 nm), then increasing again at long distances, but always with negative values. The nearly identical curves for the dodecyl sulfate potential are a consequence of no appreciable change in the micelle shape due to the limited amount of surfactant ions in the system, as discussed before.

Examining the neutral species, water and urea, also reveals an intriguing pattern of electrostatic potential. In the case of urea, despite the minimal variation in electrostatic potential, a positive peak appears near the micelle surface (ca. r = 2 nm), indicating a slight orientation of the amino groups toward the negatively charged sulfate headgroups, in agreement with the orientation curves (Figure 11a). In the outside aggregate region but also near the micelle surface (ca. *r* = 2.5 nm), we see that the electrostatic potential of urea now presents a negative peak, indicating that the partially negatively charged oxygen atoms are accumulated, which can be a consequence of the high density of cations in this region. In the bulk phase, the curve oscillates around 0, indicating the region where the urea molecules are no longer oriented preferentially. The electrostatic potential of water is similar to that of urea but slightly shifted to longer distances, with the positive peak appearing around *r* = 2.3 nm. However, as the number of water molecules varies across different systems, the height of the positive peak increases with a higher molar fraction of water. In the case of SDS in pure water, its behavior differs from that in systems containing reline. In pure water, the positive peak is significantly more intense, and there is no negative peak, resulting in a curve that declines toward the bulk phase. All of these components contribute to the overall electrostatic behavior, demonstrating that anionic aggregates in the presence of reline exhibit complex patterns that differ from classical models found in the literature.

Finally, the dynamics of the solvent species as well as of the sodium cations were analyzed by means of the diffusion coefficient, computed by the linear regression of the mean square displacement (MSD) of the species as a function of time according to eq 7. As noticed, the diffusion coefficient of all species decreases significantly as the water molar fraction decreases (Table 3), as expected due to the systematic reduction of relative viscosity upon addition of water.^33^ The diffusion coefficient could not be obtained for the surfactant since the MSD curves did not achieve a clear linear diffusive regime within the simulation time scale. Still, the comparison of MSD curves for the dodecyl sulfate anions also displays clearly a slower dynamics in the reline/water mixtures than in pure water (Figure S6 in the Supporting Information file). The slower dynamics at a water molar fraction of 0.4 resulted in the larger statistical error in the PMF calculations (Figure 3), even with longer simulations performed for each sampling window for this system.

7 $$D=\frac{1}{6}\frac{dMSD}{dt}$$

**Table 3:** Diffusion Coefficients (D) for Each Species Present in the Model Systems at 323.15 K Except the Surfactant for Which a Linear Plot in the MSD Analyses Was Not Obtained (Figure S6 in the Supporting Information File)^a^

water molar fraction at system	diffusion coefficient (10^–8^ cm^2^/s)				
	sodium	water	urea	choline	chloride
1	1040	3970	-	-	-
0.73	206	763	314	195	357
0.54	61	273	88	49	103
0.40	9	81	18	9	20

a The standard deviation was not reported because its value was negligible (approximately zero).

Water always presents the fastest dynamics at any given solvent composition, followed by the chloride and the urea. The cations sodium and choline display very similar diffusion coefficients, and the slowest diffusion without counting the surfactant. In the recent work of Voroshylova et al.,^34^ molecular dynamics simulations of SDS micelles in reline resulted in diffusion coefficients of the order of 10^–11^ cm^2^/s or smaller for choline and chloride ions in different DES at 298.15 K, indicating the dramatic effect the presence of water and the higher temperature employed in our work presents in the diffusion coefficients.

## 4 Conclusions

PMF calculations for the removal of a dodecyl sulfate monomer from the micelle indicated that the aggregation free energy for the SDS is more negative in reline/water mixtures compared to pure water, suggesting that these anionic micelles are more stable in the presence of the DES. Furthermore, PMF calculations for removing the surfactant’s head confirmed that this stability results from the stabilization effect of the charged headgroups, so that the head contribution to the total free energy of aggregation in the presence of reline becomes slightly negative, while it is positive in pure water. Analysis of the solvent structure revealed a high concentration of cations at the micelle surface in reline systems, which stabilizes the negatively charged headgroups and, consequently, the micelle. On the other hand, as the concentration of reline increases, there is a significant decrease in the number of water molecules surrounding dodecyl sulfate. This reduction is responsible for the diminished hydrophobic effect, which is one of the main driving forces behind the aggregation of structures such as micelles. However, the PMF results indicate that the stabilizing effect of the heads surpasses the reduction in the hydrophobic effect, thereby making micelles in reline/water mixtures more stable. This also helps to explain why the opposite trend is noticed in the experimental CMC values for neutral and zwitterionic surfactants, for which the shielding of the headgroup repulsion by the choline cation may not be so significant, and the decrease in the hydrophobic effect may be the dominant effect, leading to a reduction of the micelle thermodynamic stability.

Analysis of the number of molecules surrounding both the micelle and the pulled monomer revealed that, in reline/water systems, sodium—the natural counterion of dodecyl sulfate—does not have a preferential interaction. Instead, urea and choline are found in greater quantities on the micelle surface. The electrostatic potential profiles reveal that classical electrical double layer models fail to describe ion distributions around charged particles and surfaces in reline/water systems, highlighting their inadequacy for complex systems with high ionic content, such as DESs. At long distances, the total electrostatic potential in reline/water systems oscillates around zero, with increasing amplitude at higher reline concentrations. Consistently, the water and urea orientation profiles reveal reduced preferential alignment of water hydrogen atoms and urea amino groups toward the aggregate. These effects also arise from the elevated cation concentration surrounding the micelle, which screens and neutralizes its negative potential at long-range. Although no substantial changes in micelle structure were observed among the systems, these results are consistent with experimental evidence suggesting that micelles in reline are larger due to a decrease in interaggregate electrostatic repulsion. Additionally, a comprehensive evaluation of such structural effects is limited by the finite number of monomers (60) and the presence of only one preassembled aggregate in the simulated system.

Finally, the analyses presented in this work reveal oscillatory behavior and domain alternation patterns, indicating that the simulated systems deviate substantially from the ideal behavior and exhibit a high degree of complexity. Collectively, these findings enhance our understanding of the stability and structural properties of anionic aggregates in DESs, such as reline.

## Supplementary Material



## Notes

Partial charge and Lennard–Jones parameters used, RDF of S and Na atoms around micelle center of mass, sampling histograms from PMF calculations, accumulated charge (total and contributions) curves, and MSD of dodecyl sulfate ions (PDF)

## Abbreviations

SDS: sodium dodecyl sulfate
ChDS: choline dodecyl sulfate
CMC: critical micellar concentration
PMF: potential of mean force
PME: particle mesh ewald
MSD: mean square displacement
CN: cumulative number
RDF: radial distribution function
