# Supplementary material for: Stability of Sodium Dodecyl Sulfate Micelles in Reline/Water Mixtures: A Molecular Dynamics Study
Source: ACS Omega. 2026 Aug 19;11(34):50965–79. doi: 10.1021/acsomega.6c02912 (PMC13625139; doi:10.1021/acsomega.6c02912)
Supplement: Supplementary file 1 [file ao6c02912_si_001.pdf]

Supporting Information for

Stability of SDS Micelles in Reline / Water Mixtures: a Molecular Dynamics Study

*Rafaela Eliasquevici, Kalil Bernardino**

Laboratório de Química Computacional, Departamento de Química, Universidade Federal de São Carlos, Rod. Washington Luiz S/n, 13565-905 São Carlos, Brazil

Contents:

1. Tables including the partial charges and Lennard-Jones parameters for all species present in the simulations and for dihedral angles of the choline cation which were reparametrized. S3
2. Radial distribution function g(r) between the center of mass of the aggregate and selected atoms used to define the micelle surface and counterion distribuction. S6
3. Sampling histograms for the Potential of Mean Force calculations across all radial distances, computed using the Umbrella Sampling technique. S7
4. Potential of mean force curves computed using only 1/5 of the respective umbrella sampling simulations production data in order to estimate statistical errors associated with the dodecyl sulfate and methyl sulfate PMFs in Figures 2 and 3 using the bootstrap method as implemented in Gromacs. S8
5. Total accumulated charge around the micelle center of mass (defined in Equation 5 of the main text and used to compute the electrostatic potential as in Equation 6). S9
6. Accumulated charge from each chemical species around the micelle center of mass. S10
7. Mean square displacement (MSD) profiles for dodecyl sulfate in all simulated systems. S11
8. Radial distribution function (RDF) of the sodium cation around the oxygen atom of the surfactant headgroup for the equilibrium simulations of the micelles in water and reline / water mixture. S12
9. Cumulative number (CN) curves of the oxygen atoms of both urea and water around the center of mass of the aggregate obtained from equilibrium simulations in pure water and in reline / water mixtures and density of oxygen atoms as a function of the distance from micelle center of mass S13
10. Tables reporting the CN values of urea and water at the micelle surface (r = 1.5 nm), along with the radius distances at which the CN and RDF reach a value of 0.5. S15
11. RDFs of water oxygen atoms around a methane molecule, obtained from simulations containing a single methane molecule in the solvent boxes. A complementary table reports the average number of hydrogen bonds involving molecules in the first solvation shell of methane in those simulations. S16

Table S1 – Partial charges and Lennard-Jones parameters for dodecyl sulfate.

Atom	q (e)	ε (kJ/mol)	σ (nm)
S	1.284	1.046000	0.355
O (bonded only to S)	-0.654	0.711280	0.296
O (C-O-S)	-0.459	0.711280	0.300
C (from CH_2 bonded to head)	0.017	0.276144	0.350
C (from other CH_2)	-0.120	0.276144	0.350
C (from terminal CH_3)	-0.180	0.276144	0.350
H	0.060	0.125520	0.250

Table S2 – Partial charges and Lennard-Jones parameters for choline.

Atom	q (e)	ε (kJ/mol)	σ (nm)
O	-0.683	0.711280	0.312
N	-0.008	0.711280	0.325
C (CH_3 bonded to N)	0.090	0.276144	0.350
C (CH_2 bonded to N)	0.150	0.276144	0.350
C (CH_2 bonded to O)	0.145	0.276144	0.350
H (bonded to C)	0.060	0.125520	0.250
H (bonded to O)	0.418	0.000000	0.000

Table S3 – Partial charges and Lennard-Jones parameters for urea.

Atom	q (e)	ε (kJ/mol)	σ (nm)
C	0.142	0.43932	0.375
N	-0.542	0.71128	0.325
O	-0.390	0.87864	0.296
H	0.333	0.00000	0.000

Table S4 – Partial charges and Lennard-Jones parameters for sodium (Åqvist parameters).

Atom	q (e)	ε (kJ/mol)	σ (nm)
Na	1.000	0.011598	0.333045

Table S5 – Partial charges and Lennard-Jones parameters for chloride.

Atom	q (e)	ε (kJ/mol)	σ (nm)
Cl	-1.000	0.492833	0.441724

Table S6 – Partial charges and Lennard-Jones parameters for water (SPC model).

Atom	q (e)	ε (kJ/mol)	σ (nm)
O	-0.820	0.650194	0.316557
H	0.410	0.000000	0.000000

Table S7 – Parameters for Ryckaert-Bellemans function used to describe dihedral angles of the choline cation that were reparametrized from the original OPLS-AA force field (values in kJ/mol).

Dihedral	C_0	C_1	C_2	C_3	C_4	C_5
N-C-C-O	223.27236776	-2.43302684	2.02582148	0.76256715	0.61590863	0.38680925
C-C-O-H	83.57190464	1.84400893	-3.76911786	-4.22542943	6.64022733	3.14606529

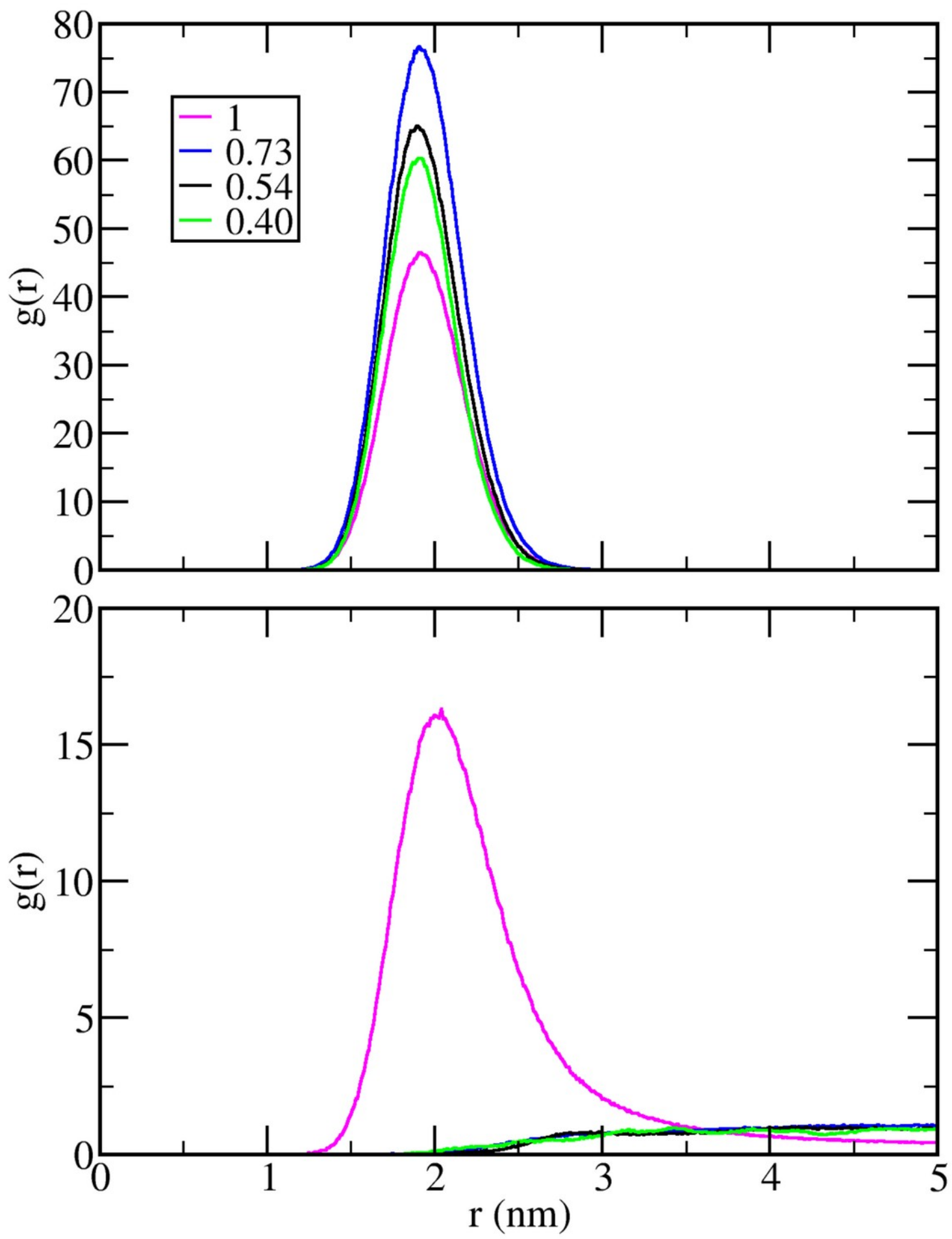

Figure S1 – TOP: Radial distribution function of the sulfur atom of the surfactant headgroup around the center of mass of the micelle for the equilibrium simulations of the micelles in water and reline/water mixture. BOTTOM: Radial distribution function of the sodium cation around the center of mass of the micelle for the equilibrium simulations of the micelles in water and reline/water mixture.

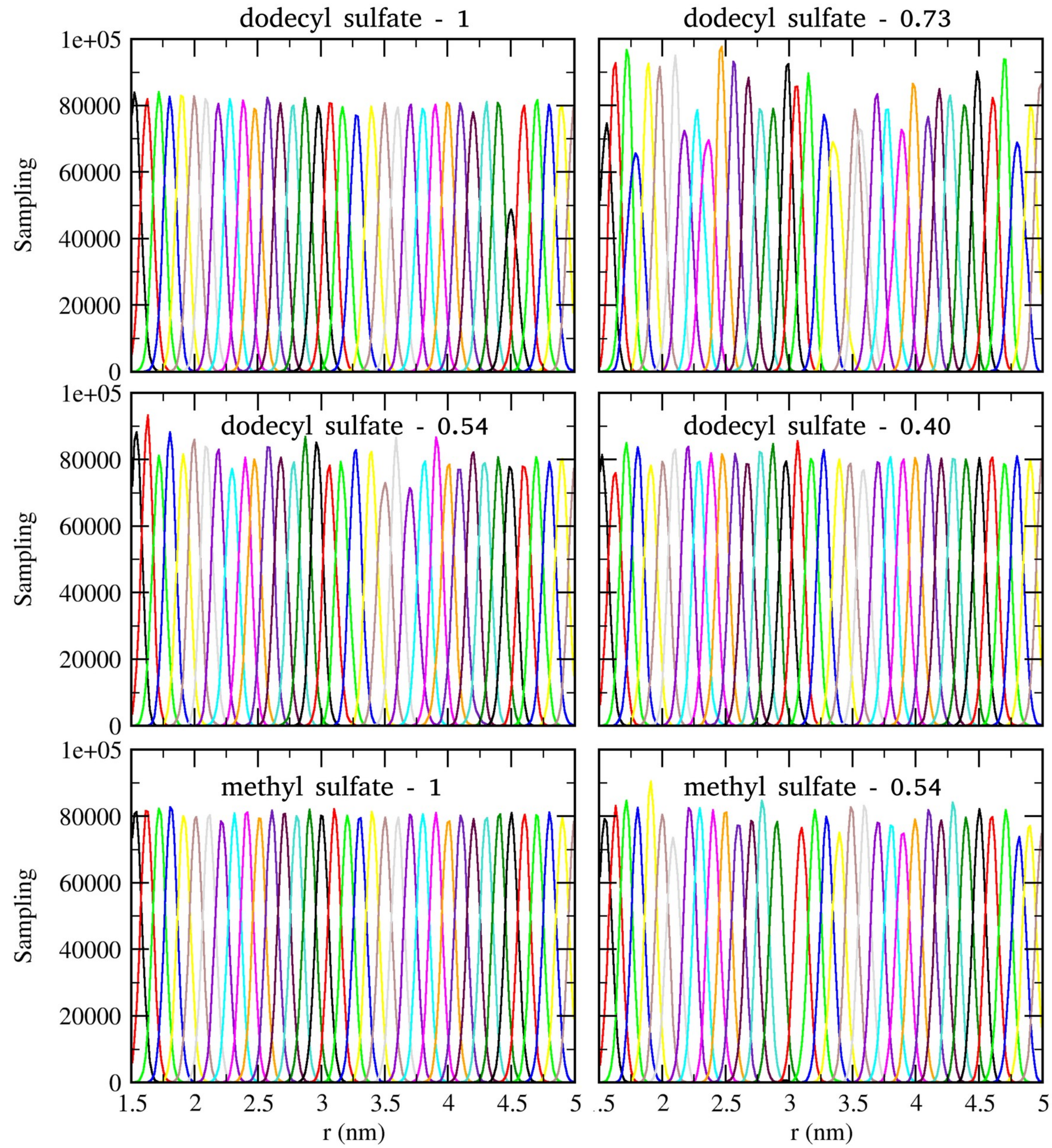

Figure S2 – Sampling histograms for the PMF calculations (1–5 nm) at water molar fractions of 1.00, 0.73, 0.54, and 0.40 for dodecyl sulfate removal, and 1.00 and 0.54 for methyl sulfate removal.

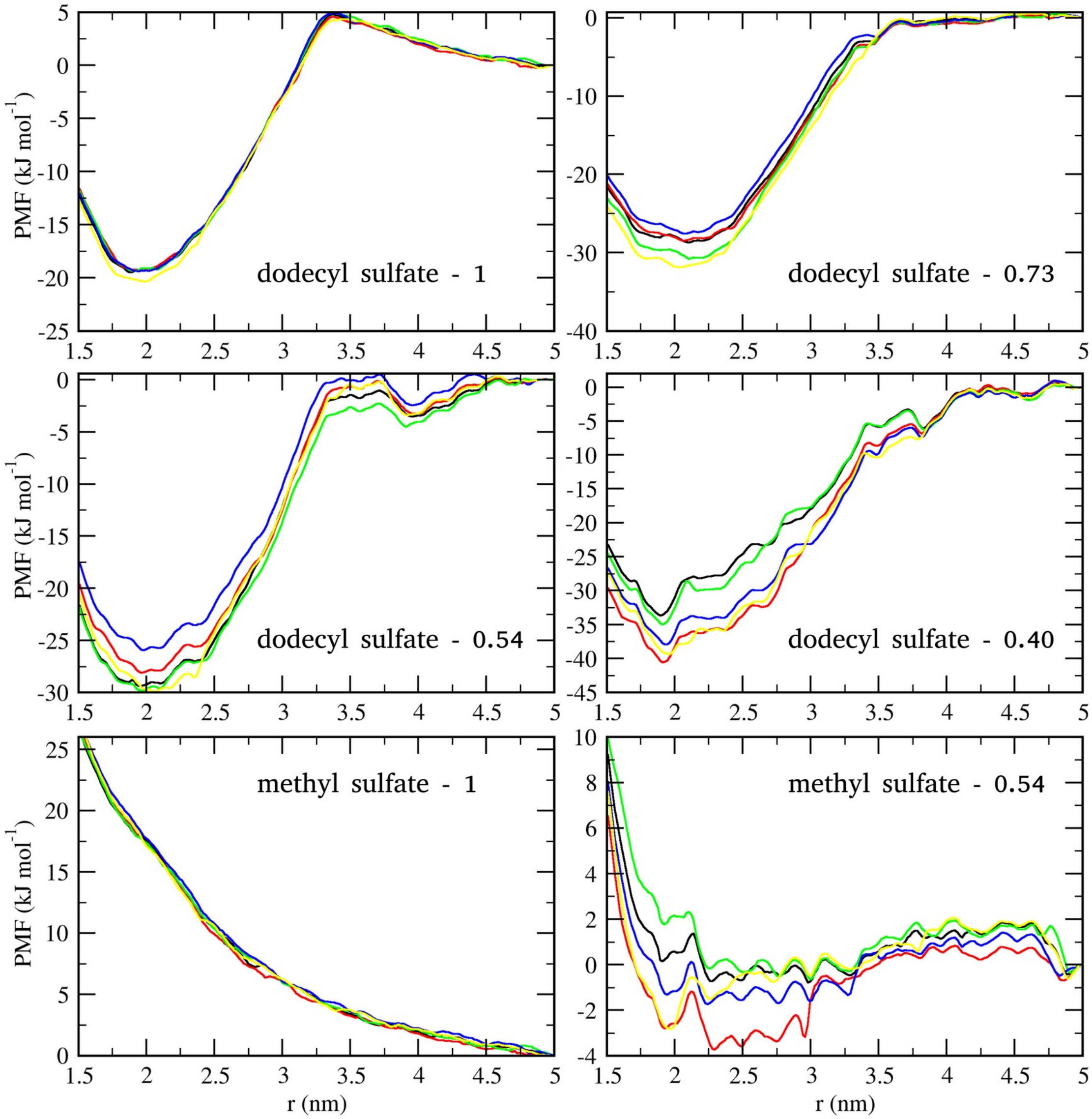

Figure S3 – Potential of mean force curves computed using only 1/5 of the respective umbrella sampling simulations production data in order to estimate statistical errors associated with the dodecyl sulfate and methyl sulfate PMFs in Figures 2 and 3 using the bootstrap method as implemented in Gromacs. Calculations were performed at water molar fractions of 1.00, 0.73, 0.54, and 0.40 for dodecyl sulfate removal, and 1.00 and 0.54 for methyl sulfate removal as indicated in each panel.

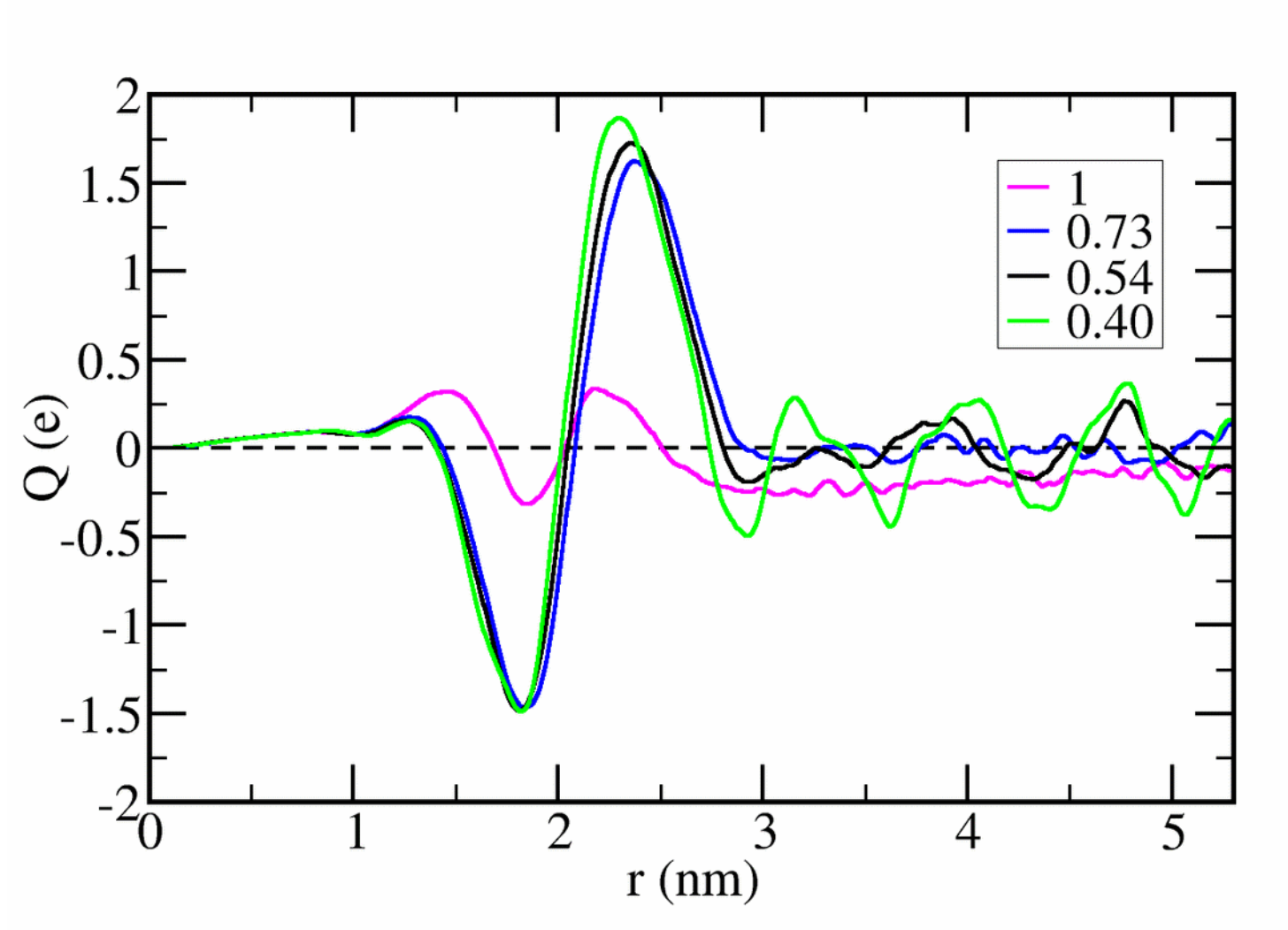

Figure S4 – Time-averaged value of the accumulated charge at each distance r from the micelle center of mass for the equilibrium simulations of the micelles in water and reline/water mixture.

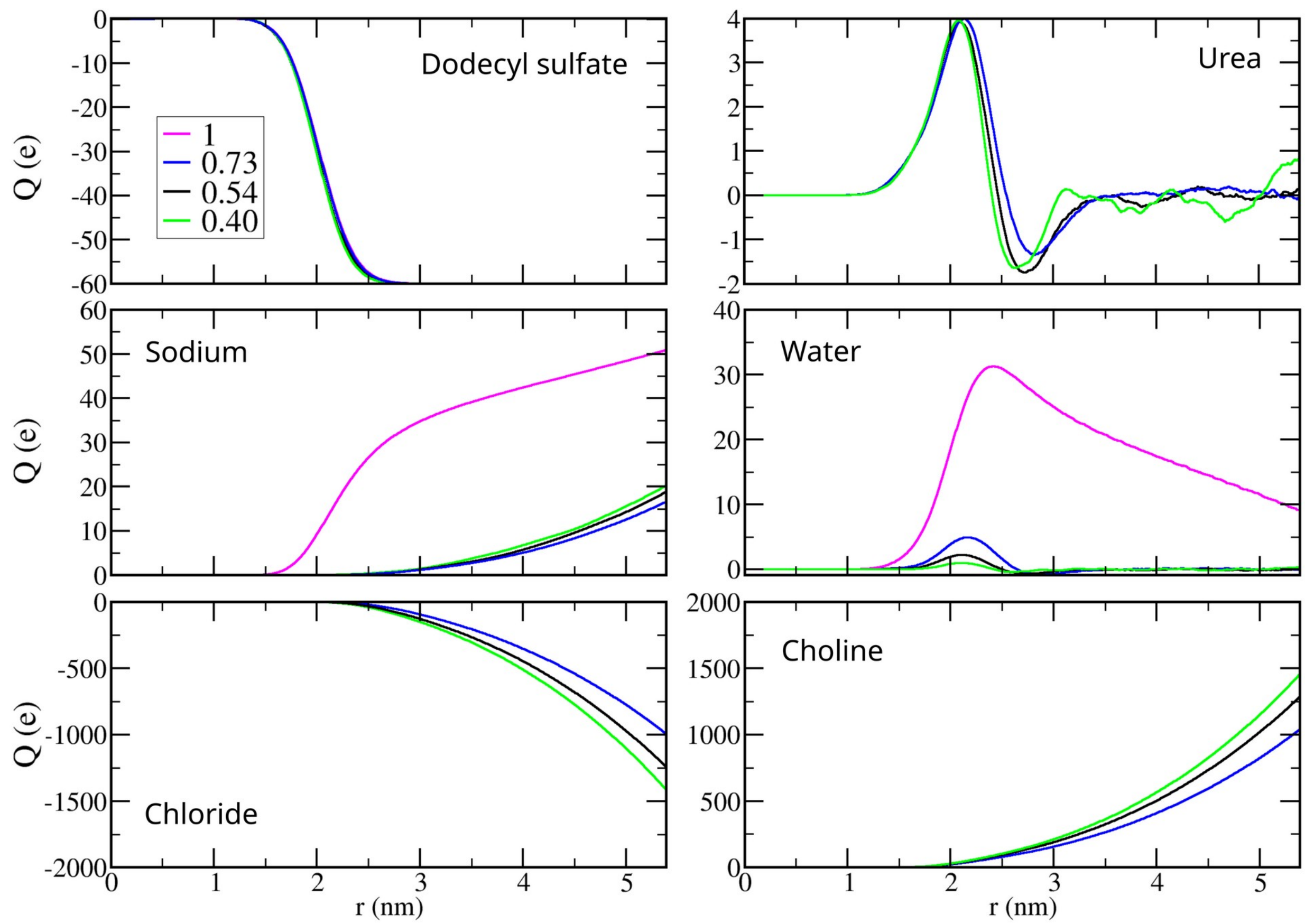

Figure S5 – Time-averaged value of the contributions of each molecule or ion presented in the system for the accumulated charge at each distance r from the micelle center of mass for the equilibrium simulations of the micelles in water and reline/water mixture.

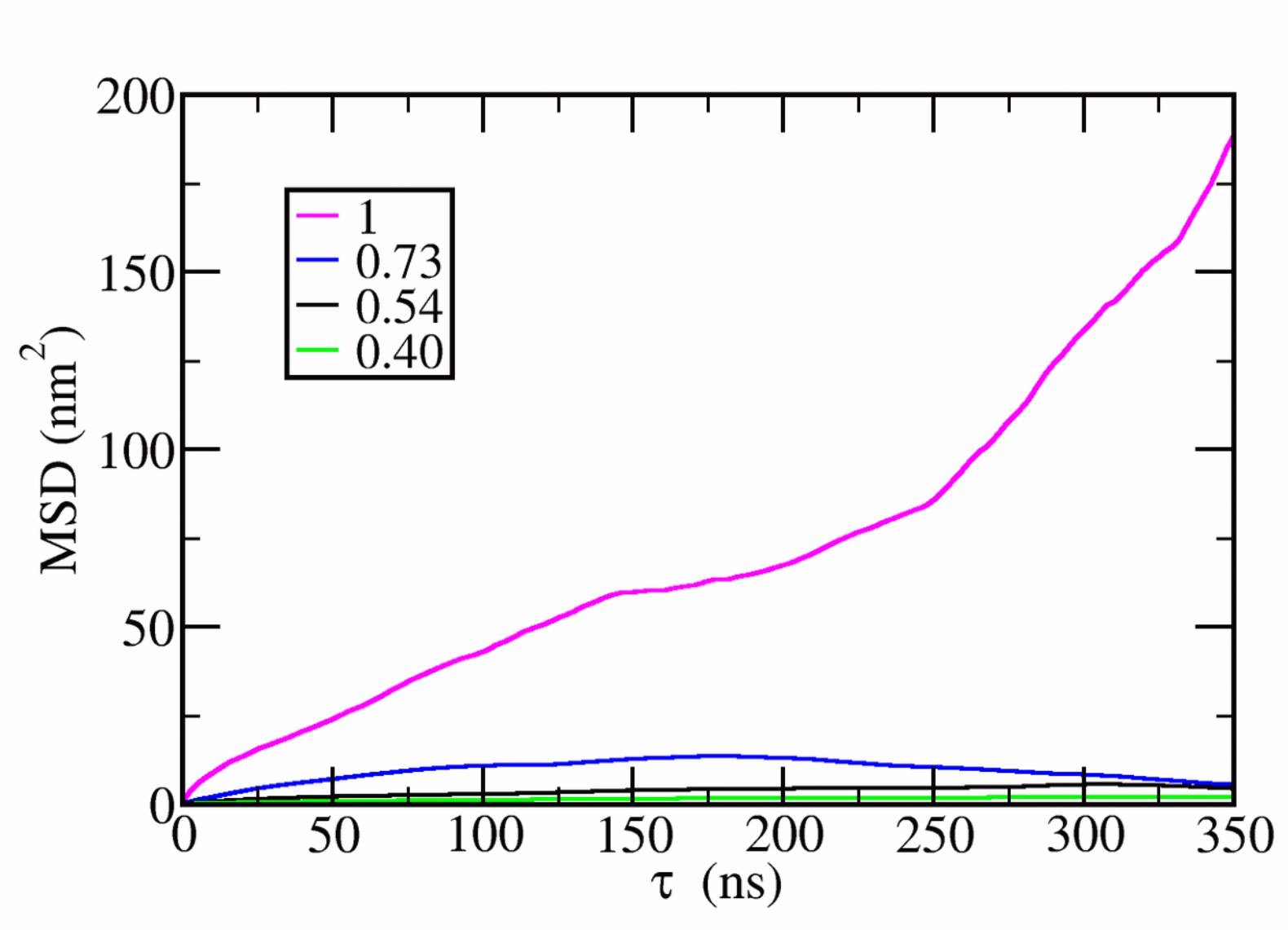

Figure S6 – Mean square displacement (MSD) profiles for dodecyl sulfate for the equilibrium simulations of the micelles in water and reline/water mixture.

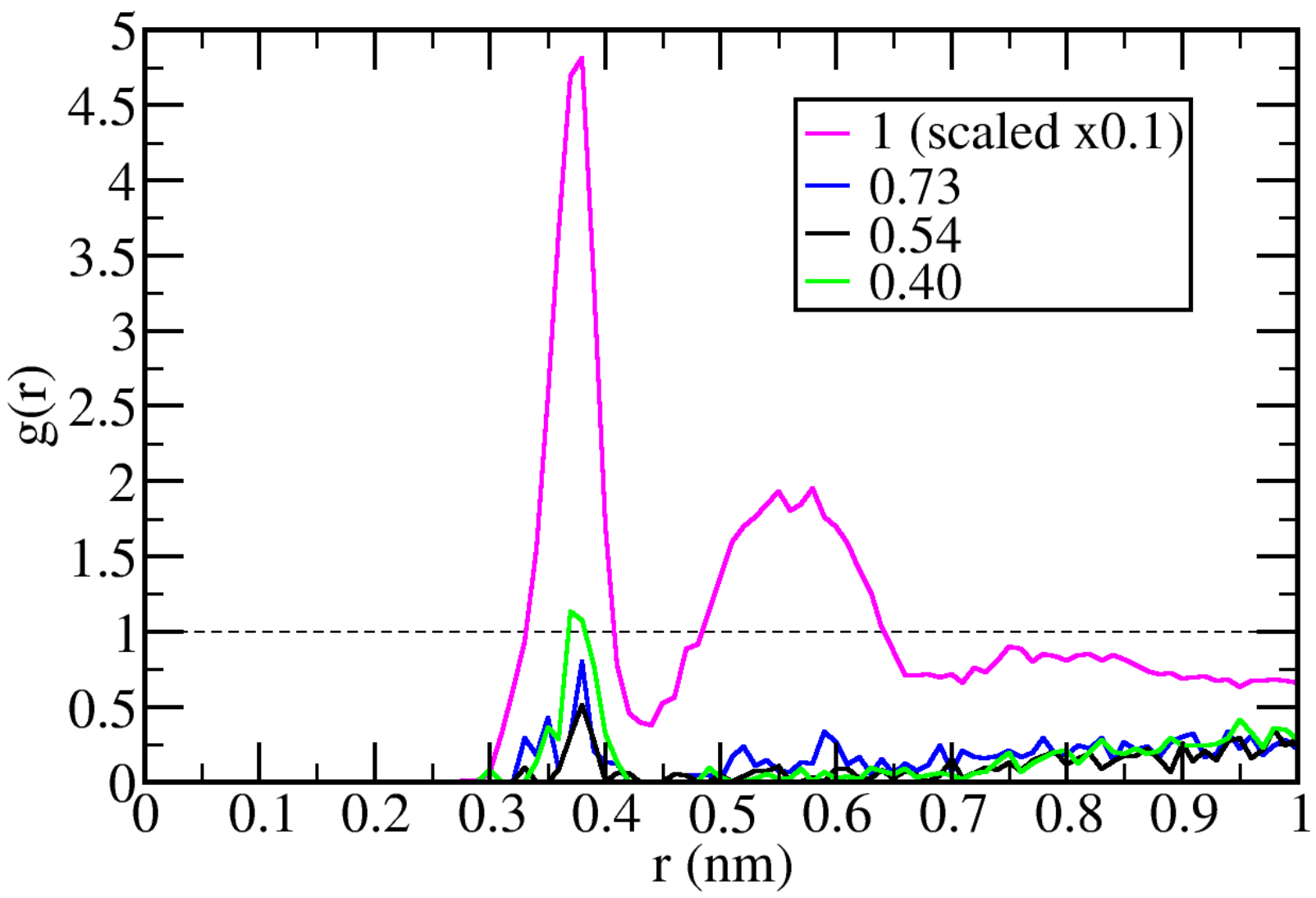

Figure S7 – Radial distribution function of the sodium cation around the sulfur atom of the surfactant headgroup for the equilibrium simulations of the micelles in water and reline / water mixture. For better visualization, the pure water curve was scaled by a factor of 0.1.

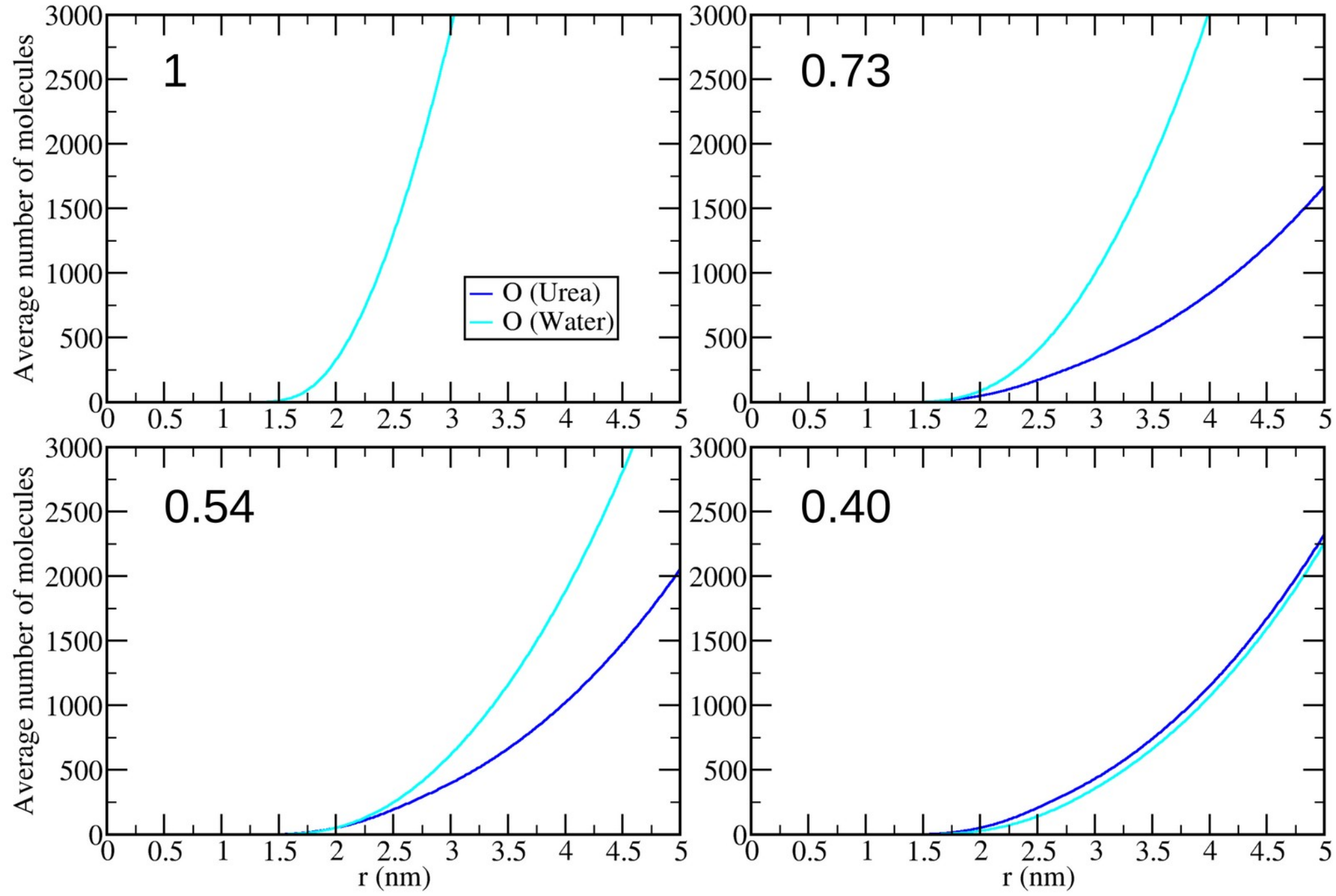

Figure S8 – Cumulative number curves of the oxygen atoms of both urea (blue curve) and water (cyan curve) around the center of mass of the aggregate obtained from equilibrium simulations in pure water and in reline / water mixtures.

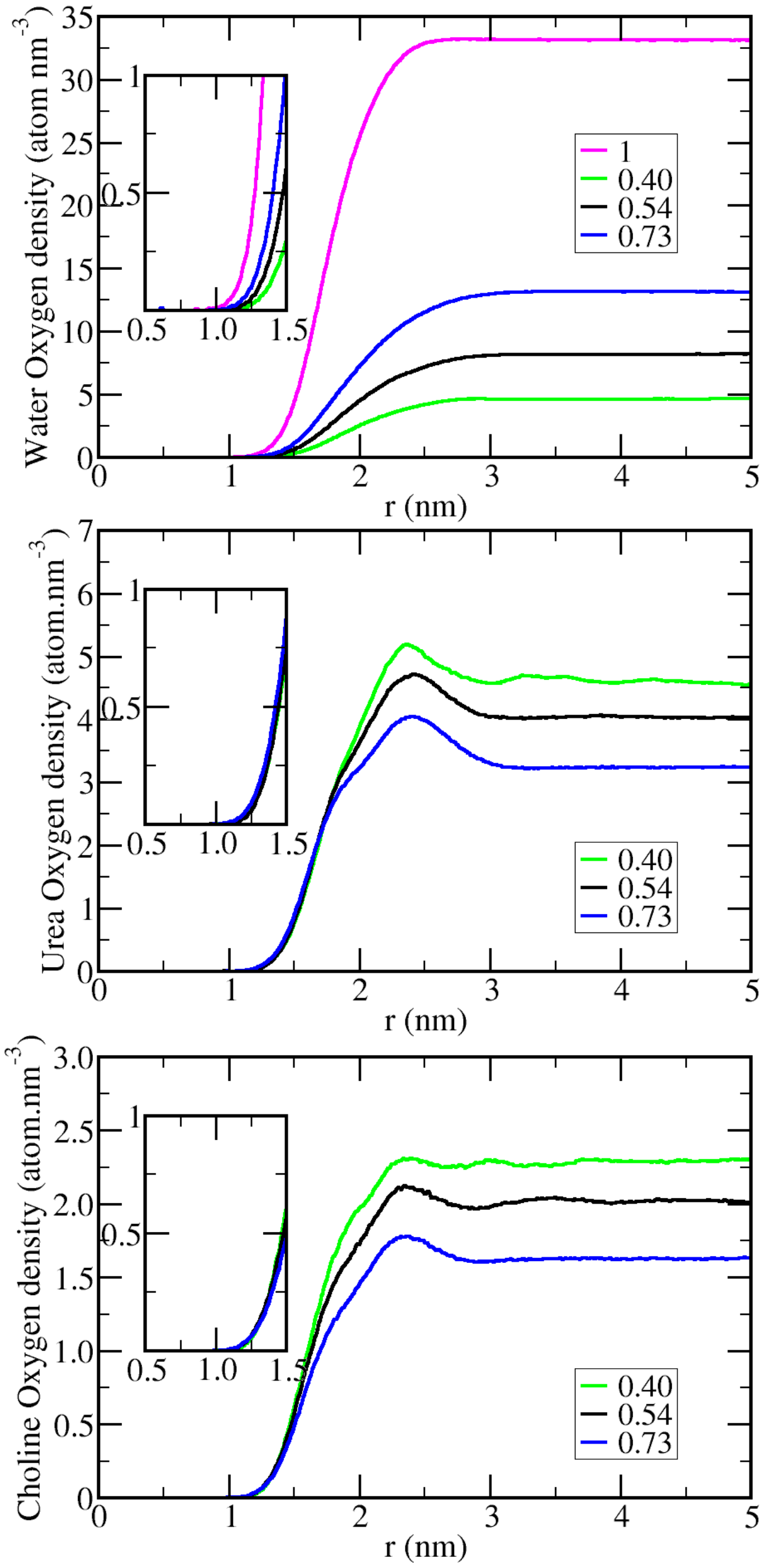

Figure S9 – Density of oxygen atoms of water (top), urea (middle) and choline (bottom) at spherical shells with different radius *r* centered on the micelle center of mass. Different colors stands for the water molar fraction in each system and insets zoom around 1.0 nm distance from the micelle center of mass.

Table S8 – Cumulative number of oxygen atoms from both urea and water around the aggregate center of mass within a radius of r = 1.5 nm, obtained from equilibrium simulations in pure water and in reline / water mixtures.

	cn (r = 1.5 nm)	
Water molar fraction in the system	O (Urea)	O (Water)
1	-	11.7
0.73	2.5	3.0
0.54	2.1	1.6
0.40	2.1	0.8

Table S9 – Distances from the aggregate center of mass corresponding to a value of 0.5 in the cumulative number and radial distribution functions for oxygen atoms from both urea and water, obtained from equilibrium simulations in pure water and reline / water mixtures.

	r (cn = 0.5) (nm)		r (rdf = 0.5) (nm)	
Water molar fraction in the system	O (Urea)	O (Water)	O (Urea)	O (Water)
1	-	1.26	-	1.77
0.73	1.36	1.35	1.61	1.94
0.54	1.38	1.40	1.67	1.94
0.40	1.38	1.46	1.72	1.95

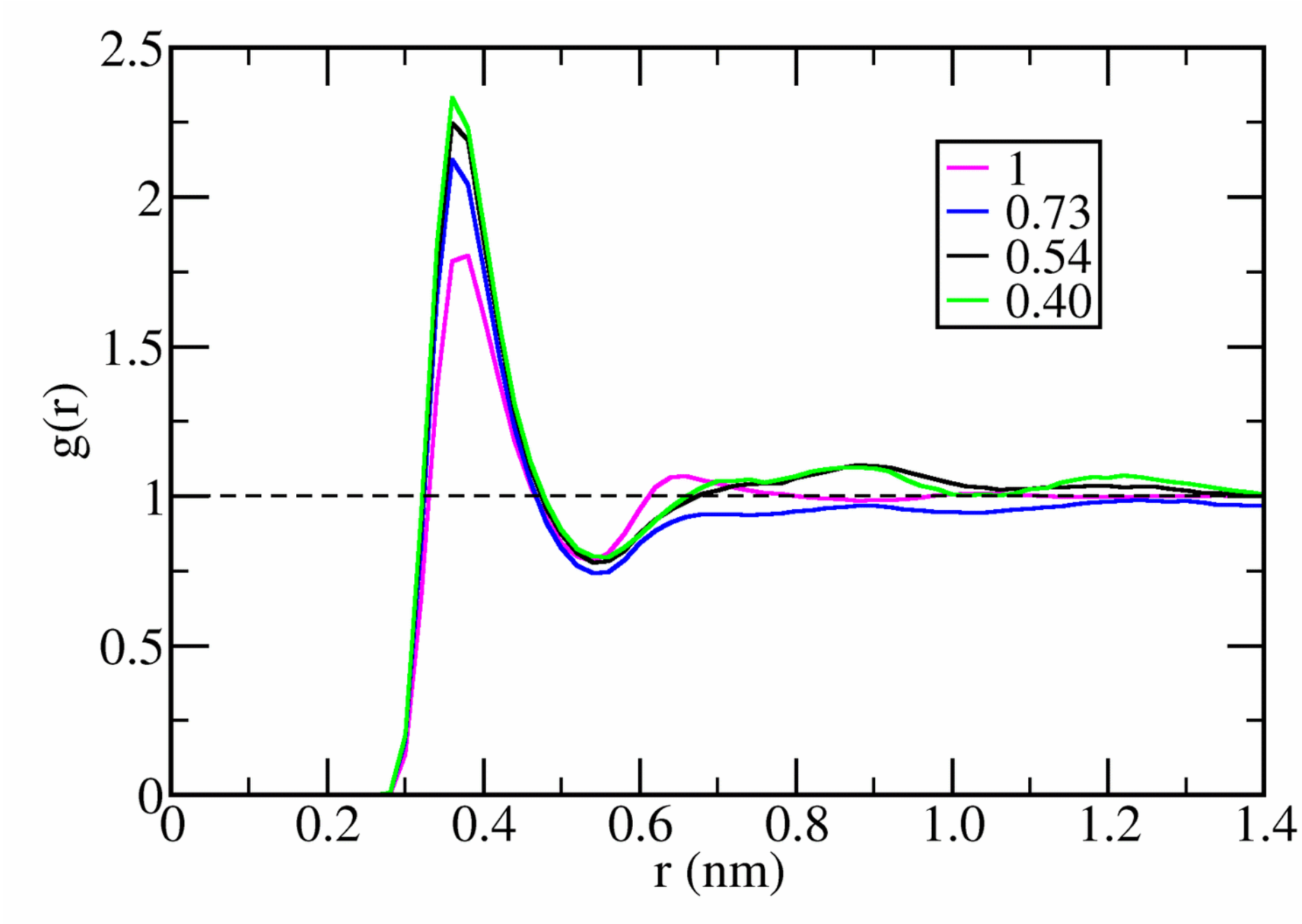

Figure S10 – Radial distribution function of water oxygen atom around methane molecule in the simulations with a single methane molecule in the solvent boxes. Different colors stands for different water molar fraction in water/reline mixtures.

Table S10 – Average number of hydrogen bonds involving molecules in the first shell of methane in pure water and reline / water mixtures with different water molar fractions.

Water molar fraction in the system	Water-water	Water-urea	Water-choline	Urea-urea	Urea-choline	Choline-choline	Total
1	30.0	0	0	0	0	0	30.0
0.73	7.7	5.1	0.8	1.9	0.4	0.0	16.0
0.54	3.9	3.7	0.9	2.1	0.6	0.1	11.2
0.40	1.4	2.9	0.5	2.4	0.7	0.1	8.0
